# Development of Self-Assembled Protein Nanocage Spatially Functionalized with HA Stalk as a Broadly Cross-Reactive Influenza Vaccine Platform

**DOI:** 10.1021/acsnano.3c07669

**Published:** 2023-12-12

**Authors:** Jaeyoung Park, Julie A. Champion

**Affiliations:** School of Chemical and Biomolecular Engineering, Georgia Institute of Technology, 950 Atlantic Dr. NW, Atlanta, Georgia 30332-2000, United States

**Keywords:** Cross-reactive influenza vaccine, Nanoparticle, Self-assembled protein nanocage, Simulation, Subunit vaccine

## Abstract

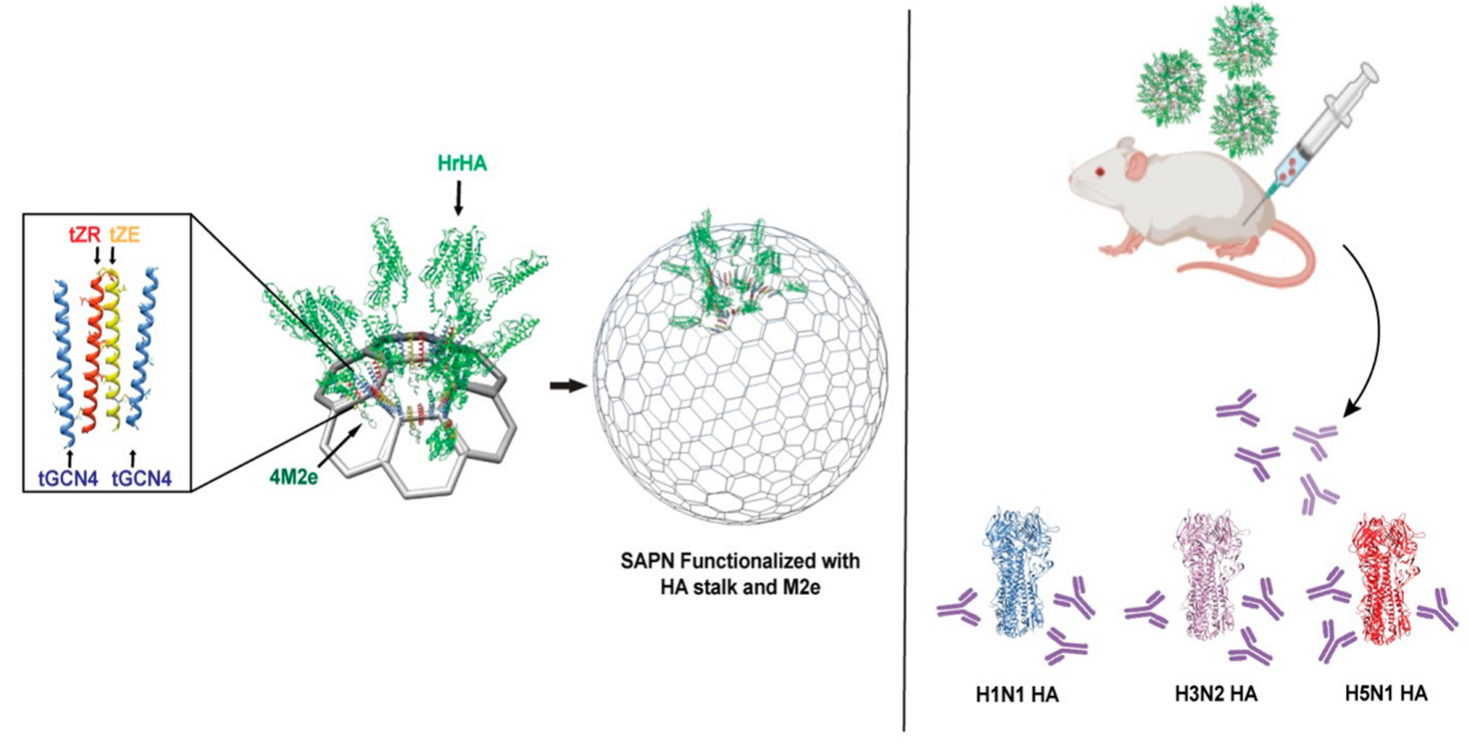

There remains a need for the development of a universal influenza vaccine, as current seasonal influenza vaccines exhibit limited protection against mismatched, mutated, or pandemic influenza viruses. A desirable approach to developing an effective universal influenza vaccine is the incorporation of highly conserved antigens in a multivalent scaffold that enhances their immunogenicity. Here, we develop a broadly cross-reactive influenza vaccine by functionalizing self-assembled protein nanocages (SAPNs) with multiple copies of the hemagglutinin stalk on the outer surface and matrix protein 2 ectodomain on the inner surface. SAPNs were generated by engineering short coiled coils, and the design was simulated by MD GROMACS. Due to the short sequences, off-target immune responses against empty SAPN scaffolds were not seen in immunized mice. Vaccination with the multivalent SAPNs induces high levels of broadly cross-reactive antibodies of only external antigens, demonstrating tight spatial control over the designed antigen placement. This work demonstrates the use of SAPNs as a potential influenza vaccine.

## Introduction

Influenza A has been one of the major global threats to human health since a zoonotic H1N1 influenza pandemic killed over 50 million people in 1918.^1^ While many strategies have been generated to curb infection, vaccination has provided the most effective protection against influenza across the world.^2^ The development of vaccines is a challenging task, especially due in part to frequent reassortment and mutation of genes to generate different influenza strains, leading to the unsuccessful design of vaccine platforms suitable for broad protection against different strains of influenza. Although seasonal influenza vaccines are developed annually, the overall effectiveness has varied significantly and remains only 10–60%.^3,4^ Therefore, the development of a universal influenza vaccine with high efficacy is needed to address issues associated with insufficient and fluctuating seasonal vaccine effectiveness as well as the antigenic variation of influenza that can lead to a pandemic.

To address the issues associated with antigenic alteration underlying the failure of seasonal vaccines, highly conserved regions of virus are considered as subunits for vaccine development. By the expression and fabrication of mixtures of conserved antigens, vaccines can be designed to provide cross-protection against various virus strains. Previously, both hemagglutinin (HA) stalk and ectodomain of matrix protein 2 (M2e) have been identified as suitable candidates for universal influenza vaccines.^5−11^ While highly conserved influenza antigens can be incorporated into subunit vaccines, the antigens are immuno-subdominant and often less immunogenic than those that are prone to mutations. The main challenge, therefore, is developing a vaccine platform that can present conserved but weakly immunogenic antigens in a way that enhances their immunogenicity to fully activate the adaptive immune system. Different approaches have been taken to design vaccine platforms that can improve the immunogenicity. Presenting multiple copies of antigens in a highly repetitive array is one of the most effective approaches to enhance immune responses.^12,13^ B cell activation, in particular, is significantly affected by the valency of vaccine nanoparticles because clustering of B cell receptors by antigens is required for initiating and inducing signaling cascades. It is also important to present antigens in their native conformations. B cell receptors and antibodies can recognize discontinuous epitopes, which are brought close to one another if and only if the protein is properly folded. We previously reported that the structure of antigens in nanoparticles is critical for humoral immune response, and solvent-mediated assembly of nanoparticles such as desolvation can disrupt antigen structure, resulting in markedly diminished humoral immunity against conformational antigens.^14^ An attractive alternative to solvent-mediated synthesis of nanoparticles is virus-like particles (VLPs) due to their ability to self-assemble into particles that mimic the polyvalency and highly organized nanostructure of native virus.^8,15−18^ This presentation of antigens in a highly dense and organized array significantly enhances humoral immune response by promoting cross-linking of B cell receptors. Also, VLP scaffolds consist of multiple antigenic components, with coat proteins exhibiting strong immunogenicity. However, the presence of heterologous antigens often results in the induction of irrelevant immune responses that can competitively inhibit immune responses against highly conserved yet poorly immunogenic target antigens. Hence, the platform may not be a desirable approach for the development of a universal influenza vaccine, which requires the effective recognition of highly conserved domains of antigens by the immune system.

Small assembling motifs such as coiled coils can be employed as building blocks of nanoparticle vaccines to minimize the off-target immune response and direct the immune response to antigens of interest while obtaining polyvalency. While coiled-coil-based nanovaccines have been very successful at presenting peptide antigens for a wide variety of vaccine applications, including infectious disease, cancer, and autoimmunity, the largest antigens reported are oligomeric small protein antigens, including tetrameric M2e and trimeric Helix C from influenza.^19−21^ A self-assembled peptide cage (SAGE) is a nanocage that utilizes synthetic homotrimeric and heterodimeric coiled coils, designated as hubs, to drive the formation of nanoparticles and can present proteins with preserved structure in a multivalent and highly organized array.^22,23^ Via site-specific fusion of proteins to the N- or C-termini of hubs, both internal and external surfaces of SAGE can be functionalized with peptides or larger oligomeric proteins, such as green fluorescent protein. Morris et al. demonstrated the use of SAGE as a subunit vaccine platform by decorating the nanocage with small ovalbumin (OVA_323–339_) or HA_518–526_ (A/PR8/34 H1N1) peptides as model antigens.^24^ When mice were immunized with peptide-decorated SAGEs, an anti-OVA_323–339_ humoral immune response was promoted. Furthermore, it was reported that HA-functionalized SAGEs significantly enhanced the response of CD8+ T cells harvested from the spleens of immunized mice. Such features of nanocages built from coiled coils can thus be utilized for the synthesis of nanoparticle vaccines, which can induce both potent humoral and cellular immune responses. While conformational B cell antigens can be presented on the highly repetitive surface of a nanoparticle, T cell epitopes do not require surface display and can be incorporated inside the nanostructure. We sought to determine whether this platform could be adapted to present large, conformational oligomeric protein antigens.

In this work, we lay out the rational design strategy for self-assembled protein nanocages (SAPNs) using engineered trimeric GCN4-pII and heterodimeric leucine zipper coiled coils and demonstrate their use as a modular vaccine for the development of cross-reactive influenza vaccine nanoparticles. To decorate the external surface of a nanocage with multiple copies of trimeric head-removed HA stalk (HrHA), it was fused to the N-terminus of GCN4-pII coiled coil, which was previously shown to effectively stabilize trimeric HA antigens and HrHA, resulting in enhanced immunogenicity.^5,25,26^ A tandem repeat of M2e epitopes derived from human, swine, avian, and fowl based on consensus sequences (4M2e)^5^ was introduced inside the nanocage to test the self-assembly design and stability *in vivo*. 4M2e was recombinantly fused to the C-terminus of a glutamic acid-rich coiled coil (ZE), a heterodimeric counterpart of an arginine-rich coiled coil (ZR); the heterodimeric leucine zippers have shown strong interactions to one another with a melting temperature above 90 °C and an affinity of 10^–15^ M and predominantly exist as a heterodimeric pair.^27−30^ The SAPNs displaying multiple copies of highly conserved antigens with preserved conformation in a controlled orientation were shown to induce broadly cross-reactive antibodies against different influenza HA subtypes, while off-target immune responses against empty SAPN scaffolds were not observed. Additionally, the lack of anti-M2e antibody response demonstrates the fidelity of the nanocage design and that M2e antigens are inside intact SAPNs.

## Results

### Design of Self-Assembled Protein Nanocage

**Figure 1:**
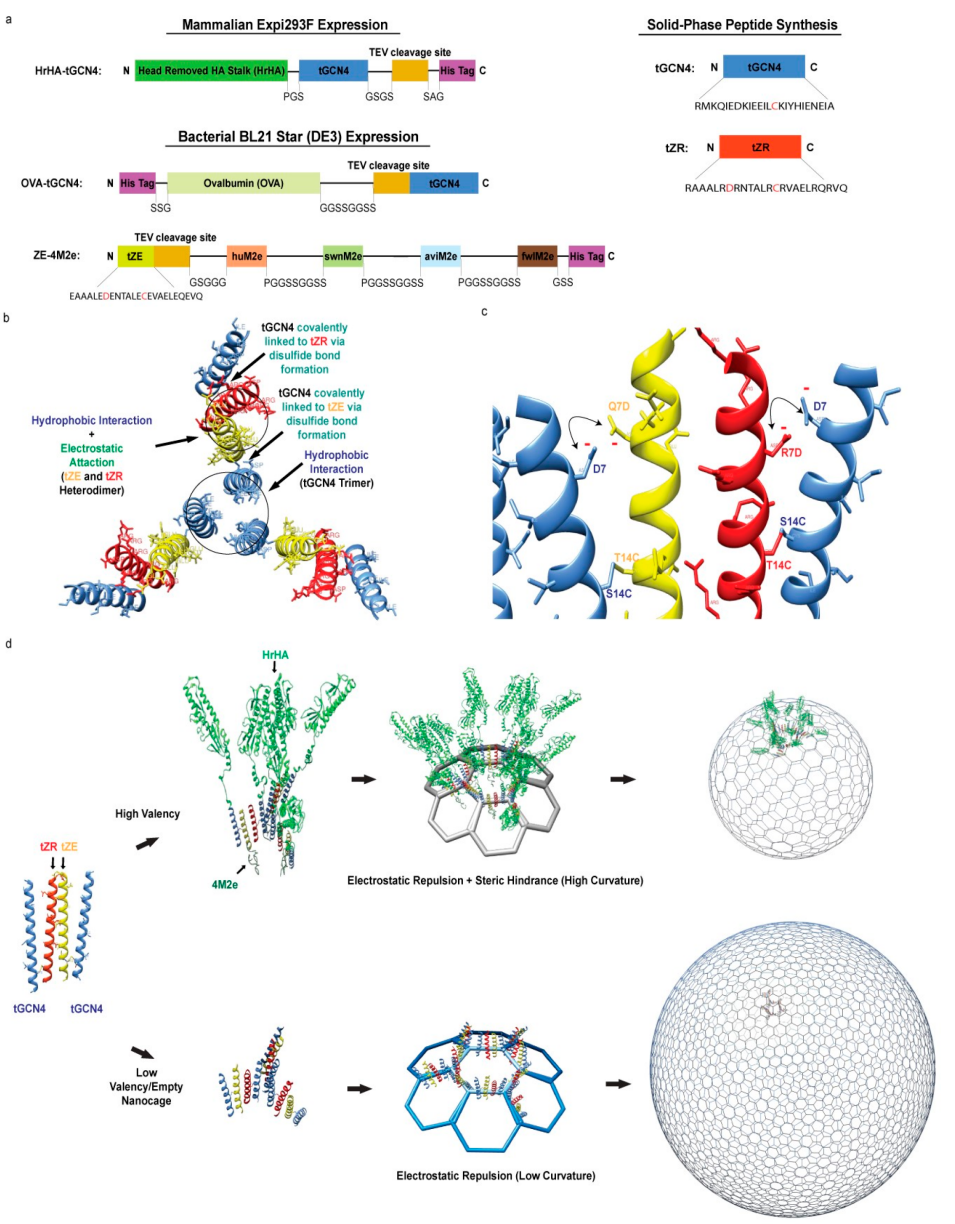
Design and assembly mechanism of a self-assembling protein nanocage. (a) Linear diagram of HrHA-tGCN4, OVA-tGCN4, tZE-4M2e, tGCN4, and tZR recombinant fusion proteins and tGCN4 and tZR synthetic peptides. The N- and C-termini are labeled as N and C, respectively. (b) Mechanism of tGCN4 (blue), tZR (red), and tZE (yellow) oligomerization. (c) Electrostatic repulsion between each motif induced between the negatively charged aspartate residue (D7) of tGCN4 and aspartate mutations in tZE (Q7D) and tZR (R7D). Cysteine mutations in tGCN4 (S14C) and tZE or tZR (T14C) covalently link one another via disulfide bonds. (d) Schematic representation of SAPN assembly. SAPN is formed by a hexagonal network established from the assembly of HrHA-tGCN4, tZE-4M2e, and tZR. 4M2e fused to the C-terminus of tZE was incorporated inside the hollow SAPN. N-terminal fusion of HrHA to trimeric tGCN4 placed trimeric HrHA on the surface of SAPN in a highly repetitive array.

Inspired by the SAGE design,^22^ we sought to adapt it to coiled coils that would support an HrHA trimeric quaternary structure and enable antigen fusion to both sets of coils. To design a spherical nanocage, short GCN4-pII (tGCN4), ZE (tZE), and ZR (tZR) motifs, which were generated by truncating each coiled coil from 33 amino acids to 24 amino acids in length, were used as SAPN modules (Figure 1a–c); each coiled coil consisted of 24 amino acids^22^ to avoid any possibility of weak curvature due to steric hindrance between long coiled coils. A cysteine mutation was introduced near the C-terminus of each coiled coil (Figure 1a–c) to connect tGCN4 to tZE (tGCN4/tZE) or tZR (tGCN4/tZR) via the formation of disulfide bonds.^22^ Cysteine was substituted for a serine residue (S14C) near the C-terminus of tGCN4 and a threonine residue (T14C) near the C-terminus of both tZE and tZR (Figure 1c). Subsequently, trimerization of tGCN4 and heterodimeric pairing of tZE and tZR of disulfide-linked tGCN4/tZE and tGCN4/tZR complexes would form a hexagonal network (Figure 1b,d). Local curvature of the network was introduced by electrostatic repulsion between aspartate residues (tGCN4 D7 and mutations tZE Q7D and tZR R7D) near the N-terminus of each coiled coil (Figure 1a–c). This, in turn, should lead to the formation of a spherical nanocage, as illustrated in Figure 1d. We also speculated the formation of smaller nanocages by functionalizing the surface of SAPN with multiple copies of antigens due to the steric hindrance between antigens on the external surface in addition to the electrostatic repulsion near the N-termini of coiled coils.

Another important component for the design of SAPN as a vaccine platform is the presentation of flu antigens in a repetitive array with controlled orientation and oligomericity. To achieve a high valency of antigens with an appropriate orientation, HrHA was recombinantly fused to the N-terminus of each tGCN4 monomer (HrHA-tGCN4), whereas 4M2e was fused to the C-terminus of tZE (tZE-4M2e). In conjunction with a curved hexagonal network, N- or C-terminus specific fusions of antigens to the motifs would form a SAPN comprising multiple HrHA antigens on the surface in a highly ordered and repetitive array and 4M2e antigens inside the nanocage. As reported by Yassine et al., the C-terminal domain of HA stalk can splay apart without stabilization.^31^ However, fusion of HrHA at the C-terminus to tGCN4 would also stabilize the trimeric conformation of HrHA and minimize splaying of the C-terminal domain to mimic the native, trimeric conformation of HA. In addition to HrHA- and 4M2e-functionalized SAPNs, a SAPN presenting full-length OVA proteins was also designed by genetically fusing OVA to the N-terminus (Figure 1a) to examine the ability to incorporate diverse antigens without compromising nanocage integrity.

### Molecular Dynamics Simulation Demonstrating the Viability of Nanocage Design

SAPN was designed by engineering trimeric GCN4 and a heterodimeric pair of ZE and ZR, yet it remained unknown whether protein engineering and binding motifs of the design would direct intended folding of proteins to form a spherical nanocage. There were mainly two aspects of the design that needed to be validated: (i) introduction of electrostatic repulsion between coiled coils near the N-terminus by negatively charged aspartate residues and (ii) trimerization of tGCN4 and dimerization of tZE and tZR following truncation and mutations. Therefore, to assess the feasibility of our SAPN design, GROMACS was utilized for MD simulations of protein folding and interaction.

To prepare a working model, the head domain of HA (PDB: 1RVX) was replaced with glycine linkers to make HrHA protein as described previously.^5^ In addition, trimeric HrHA-tGCN4 was linked to tZE via a disulfide bond (HrHA-tGCN4/tZE) and laid with three units of tGCN4/tZR complexes by using Chimera. Prior to simulation, the model system was stabilized by energy minimization and NVT and NPT equilibration. Following stabilization, the system was simulated for 500 ps using the Amber99SB-ildn force field.

**Figure 2:**
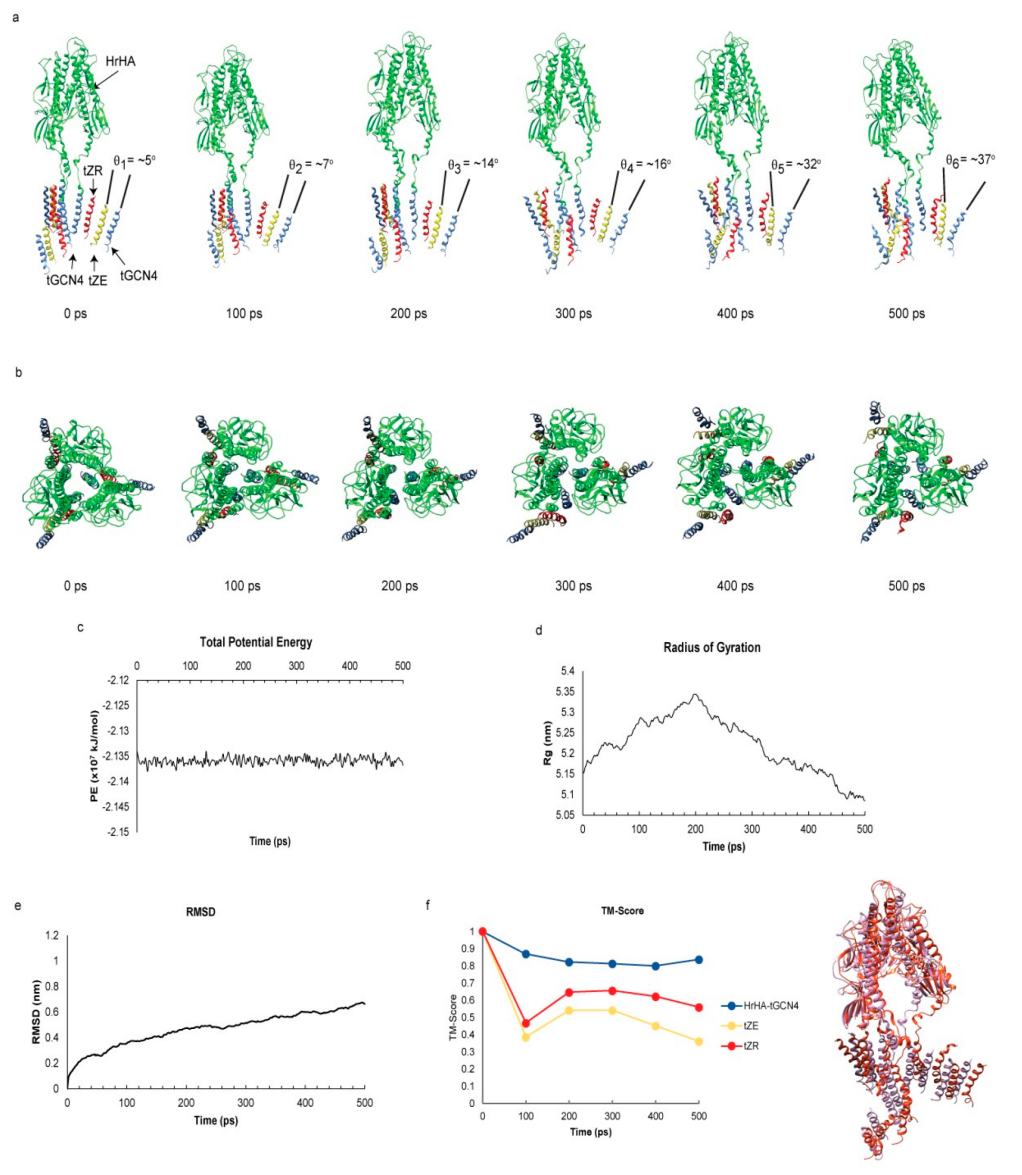
MD simulation of SAPN assembly motifs for 500 ps. (a) Side view of the working model, trimeric HrHA(green)-tGCN4(blue)/tZE(yellow) complexes with three units of tGCN4/tZR(red) complexes. Electrostatic repulsion by negatively charged aspartate residues near the N-terminus (top of motif) with disulfide bonds near the C-terminus (bottom of motif) propagated increases in angles (θ_1_, θ_2_, θ_3_, θ_4_, θ_5_, θ_6_) between the assembly motifs. (b) Top view of model showing compaction of HrHA-tGCN4 (center of protein structure) over simulation time. (c) Total potential energy of simulation system. (d) Radius of gyration indicating changes in compactness of model structure over 500 ps. (e) Root mean square deviation of the protein complexes. (f) TM-Score representing the structural similarity between simulated protein and *ab initio* reference protein model with score values between 0 and 1 indicating unmatched and perfectly matched structures, respectively (left). Structures of simulated (purple) and *ab initio* reference (red) protein structures are compared by superimposition (right).

After simulation, electrostatic repulsion between tGCN4 and tZE or tZR was observed while disulfide bonds held the peptides near one another near the C-terminus, splaying out the N-termini of the peptides as evidenced by increased angles between the peptides near the N-terminus (Figure 2a, Movie S1, and Movie S2). This suggests that the engineered motifs could induce local curvature, an essential prerequisite to form a spherical nanocage. At the same time, heterodimeric pairing of tZR and tZE driven by hydrophobic and electrostatic interactions remained intact. We next assessed whether engineered tGCN4 could still maintain its trimeric conformation. As demonstrated in Figure 2b, HrHA fused to trimeric tGCN4 became more compact, indicating that trimerization was successfully induced, and there was no evidence for splaying at the C-terminus. To ensure that the dynamics of the model was not affected by unstable energy of the system, the total potential energy of the model system was evaluated and verified to be constant (Figure 2c). Interestingly, the radius of gyration, an indicator of structure compactness, increased for the first 200 ps and then decreased during 200–500 ps (Figure 2d). Consistent with the observations shown in Figure 2a,b, the model expanded as negative charges near the N-terminus of each motif repelled one another while forces of coiled coil oligomerization became dominant and made the model more compact at 500 ps compared to the *ab initio* model structure at 0 ps.

Although the radius of gyration did not appear to be stable over 500 ps, the mean square deviation (RMSD) only slightly increased from 300 to 500 ps (Figure 2e), indicating that the folded recombinant protein was becoming stable. The template modeling (TM) score was also calculated to evaluate the structural variability between the simulated protein and the ab initio reference protein. As indicated by a TM-score >0.9 (Figure 2f),^32^ the HrHA-tGCN4 structure was very similar to the ab initio protein structure, suggesting that the protein structure was not altered by the disulfide bond or the electrostatic repulsion between tGCN4 and tZE or tZR. A TM-score for tZR >0.5 over 500 ps also represents the structural similarity to its initial protein model^32^ despite the electrostatic repulsion and its interaction with tZE. In contrast, tZE showed that the TM-score was below 0.5, suggesting that the structure was not the same as its initial structure. However, the score was 0.36, which was still higher than 0.17, implying that the structure was not significantly altered and still shared similar local folding with its initial structure.^32^ The change in the tZE structure also accounts for the slight increase in the RMSD from 300 to 500 ps. However, in concert, the MD simulation demonstrates that the engineered coiled coils did not lose their ability to induce oligomerization and could accomplish key aspects of the design for the formation of SAPNs.

### Generation and Characterization of Self-Assembled Protein Nanocage

**Figure 3:**
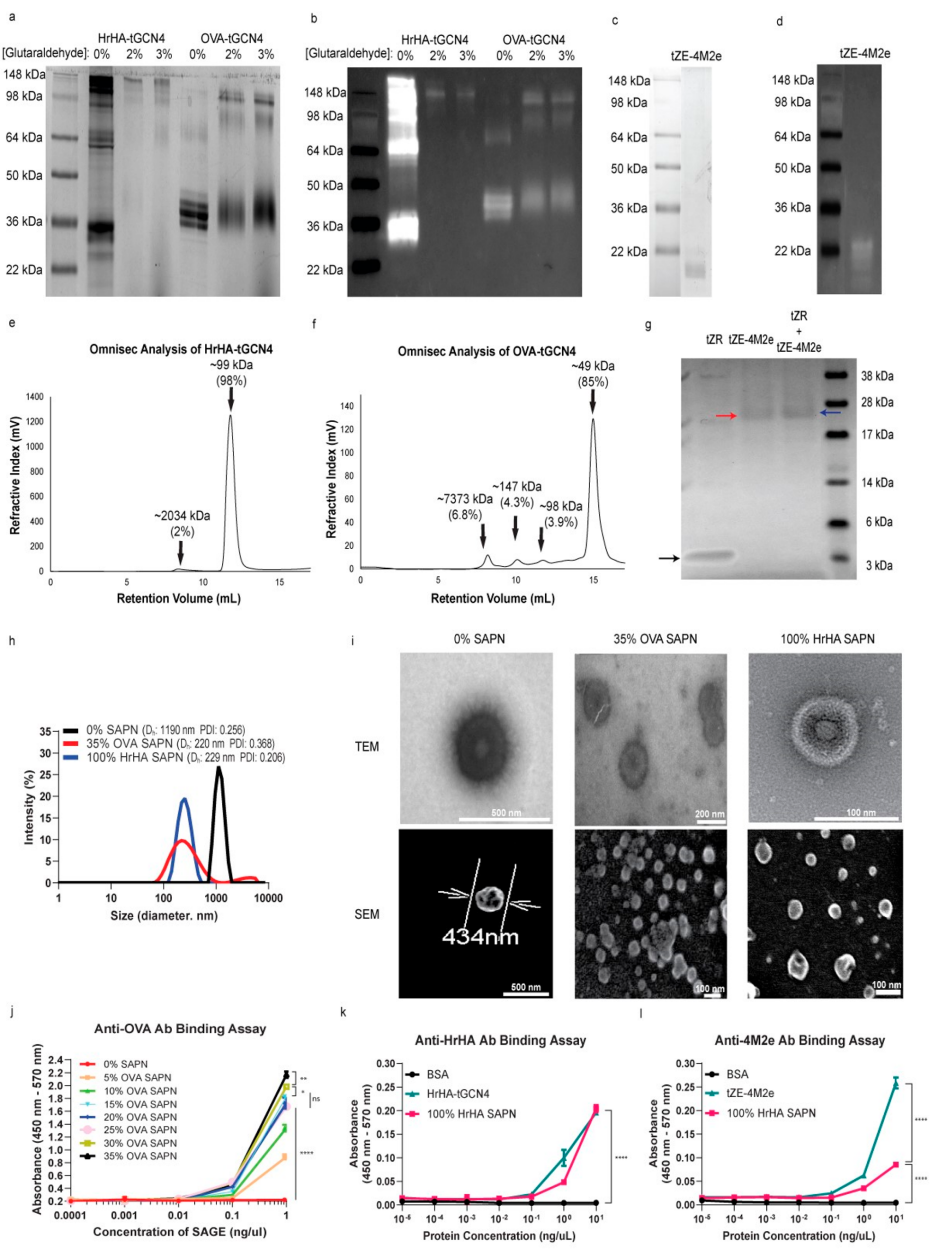
Synthesis and characterization of SAPNs. (a) SDS-PAGE (Tris-Glycine gel) and (b) Western blot of HrHA-tGCN4 and OVA-tGCN4 with and without 2% and 3% glutaraldehyde cross-linkers. (c) SDS-PAGE (Tris-Glycine gel) and (d) Western blot of tZE-4M2e. OmniSEC analysis of (e) HrHA-tGCN4 (99 kDa for trimer) and (f) OVA-tGCN4 (49 kDa for monomer; 98 kDa for dimer; 147 kDa for trimer) fractions. (g) Nu-PAGE (Bis-Tris gel) analysis of tZE-4M2e (17 kDa) and tZR (3 kDa) proteins and a mixture thereof. Monomers (∼3 kDa for tZR and ∼17 kDa for tZE-4M2e) and heterodimer (∼20 kDa) of tZR and tZE-4M2e are indicated with black, blue, and red arrows, respectively. (h) Size distribution measurement by DLS for 0% SAPNs, 35% OVA SAPNs, and 100% HrHA SAPNs. (i) TEM and SEM images of 0% SAPNs, 35% OVA SAPNs, and 100% HrHA SAPNs. (j) Anti-OVA binding assay to evaluate functional valency of 0–35% OVA SAPNs. (k)Anti-HrHA and **(**l) anti-4M2e binding assay to evaluate antigen presentation in 100% HrHA SAPNs functionalized externally and internally with HrHA and 4M2e, respectively.

After MD simulation provided predictive support for folding and designed the assembly of the engineered binding motifs, we expressed recombinant proteins and synthesized SAPNs. Recombinant HrHA-tGCN4 and OVA-tGCN4 were expressed from Expi293F mammalian cells and *E. coli* BL21 Star (DE3), respectively. To verify their expression, the purified proteins were reduced and subjected to tris-glycine SDS-PAGE followed by Western blot analysis. Eluted HrHA-tGCN4 protein displayed three bands at 33, 66, and 99 kDa, representing fractions of monomers, dimers, and trimers (Figure 3a,b). Two bands with a strong intensity at 49 kDa and a weak intensity at 98 kDa corresponding to monomers and dimers, respectively, were identified from OVA-tGCN4. Bacterial expression of monomeric tZE-4M2e with an expected molecular weight of 17 kDa was also confirmed by SDS-PAGE and Western blot (Figure 3c,d). After stabilization by irreversible 2% or 3% glutaraldehyde cross-linkers, only one fraction containing trimers was identified from HrHA-tGCN4 (Figure 3a,b), indicating that HrHA-tGCN4 consists mainly of trimers. Stabilized OVA-tGCN4 exhibited monomers, dimers, and trimers but favored monomers as a dominant form, as evidenced by the strongest intensity at 49 kDa. Subsequently, OmniSEC was employed as an orthogonal method to further assess the oligomeric state of each recombinant protein. Consistent with SDS-PAGE and Western blot, most HrHA-tGCN4 (98%) was observed at a peak with a molecular weight of 99 kDa (Figure 3e), indicating that tGCN4 promoted the trimerization of HrHA; the remaining peak area (2%) represented small fractions of aggregates with an estimated molecular weight of ∼2037 kDa. In contrast, 85% of the peak area from OVA-tGCN4 was monomers with a molecular weight of 49 kDa while the remaining fractions of OVA-tGCN4 mainly consisted of monomers and dimers (Figure 3f), likely from steric hindrance due its large molecular weight and native monomer state without inherent surface attraction. In addition, only small fractions (6.8%) of OVA-tGCN4 showed aggregation (∼7373 kDa). It is worth noting that native OVA predominantly folds in a monomeric conformation, though it can also become dimeric and rarely trimeric at high concentrations according to previous studies, resulting in three bands when it is separated by gel electrophoresis.^33,34^ This may explain why the globular ovalbumin fused to tGCN4 favored the monomeric conformation while trimerization of HrHA-tGCN4 was seen. Nu-PAGE gel analysis was performed to assess the oligomeric conformation of the tZE and tZR mixture. When the motifs were not mixed, the tZR and tZE-4M2e monomers were observed. However, the tZR band disappeared when tZR and tZE-4M2e were mixed at an equimolar concentration, as shown in Figure 3g, and pairing of tZR and tZE-4M2e resulted in a single band (∼20 kDa) with a stronger intensity than that from tZE-4M2e alone at the same loaded amount of tZE-4M2e, suggesting that major fractions of tZE and tZR exist in a heterodimeric conformation.

Next, we evaluated the secondary structure of each protein by circular dichroism (CD). The heterodimeric motifs, tZR and tZE-4M2e, exhibited characteristic minima at ∼200 nm (Figure S1a), indicating random coil structures lacking α-helical content. This observation was in alignment with CD spectra typically seen in acidic or short coiled coils when not paired with their partners.^35−38^ CD spectra of tGCN4 displayed a characteristic minimum at 222 nm, while another characteristic minimum at 208 nm was obscured. The partial loss of α-helical character might be due in part to its short length or modification with a cysteine residue. To computationally evaluate the structural change, the tGCN4 structure was first predicted by ColabFold before MD simulation. ColabFold was built on AlphaFold2, an artificial intelligent program trained on a large number of proteins from public archive of protein sequence with associated structure, and supported with a MMseqs2-based fast homology sequence searching method.^39^ The predicted tGCN4 structure consisted of α-helical coiled coils (Figure S1c). However, the structure near the cysteine residue was partially altered after MD simulation was performed for 20 ns (Figure S1d and Movie S3). Despite the effect of the cysteine residue on the structure of tGCN4, it was still able to induce trimerization of HrHA. The structure of expressed recombinant antigens was also analyzed by CD. The CD signal of the OVA-tGCN4 complex was comparable to that of the native OVA protein. When a OVA-tGCN4 monomer was complexed with two tGCN4 monomers (2:1 tGCN4:OVA-tGCN4), no significant change in the ellipticity was observed, implying that the OVA structure was not altered by trimerization of tGCN4 (Figure S1a). A ratio of 2:1 tGCN4:OVA-tGCN4 was chosen to render tGCN4 amenable to trimerization as the OVA favored monomeric conformation. HrHA-tGCN4 was characterized by its strong CD signal at 222 nm, suggesting that it consisted of mainly α-helices and partially β-sheets. ColabFold was used to computationally verify that the structure was analogous to our *ab initio* structure of HrHA-tGCN4 for MD simulation (Figure S1b), and the structure comprised a high α-helical content and a low-to-moderate content of β-sheets.

To assemble HrHA SAPNs, HrHA-tGCN4/tZE-4M2e and HrHA-tGCN4/tZR complexes were generated as disulfide linked building blocks. Prior to this, free thiol groups of tGCN4 were activated with aldrithiol-2 to ensure the asymmetric covalent bonding of tGCN4 to tZE-4M2e or tZR and not to itself. HrHA-tGCN4/tZE-4M2e and HrHA-tGCN4/tZR were then mixed at an equimolar ratio to form SAPNs with surfaces fully covered with HrHA (called 100% HrHA SAPN). Similarly, SAPNs without HrHA yet containing 4M2e inside the nanocages were prepared by mixing tGCN4/ZE-t4M2e and tGCN4/tZR modules at equimolar concentration. OVA SAPNs were formed in the same manner, but the maximum valency that they could achieve was approximately one-third of the occupation sites on tGCN4, as the majority of the OVA-tGCN4 was monomeric. OVA SAPNs with a high valency of OVA, designated as 35% OVA SAPN, were generated by mixing 35% OVA-tGCN4 and 65% tGCN4 to make trimeric coiled coil hubs, each containing approximately 1 OVA molecule. As measured by dynamic light scattering (DLS) in Figure 3h, the size of 0% SAPN was 1190 nm. When SAPNs were fully decorated with OVA (35%) or HrHA (100%) on the surface, the size decreased to 220–229 nm. The decrease in nanoparticle size might be due to the steric hindrance of antigens on the surface, increasing the curvature and forcing the formation of small nanocages. It is worthwhile to mention that electrostatic repulsion between coiled coils near the N-terminus is necessary for the formation of spherical nanocages, as demonstrated by Ross et al.^23^ Although 35% of the OVA SAPN displayed a relatively polydisperse size distribution, the size distribution of 100% HrHA SAPN was monodisperse. Negative stain transmission electron microscopy (TEM) and scanning electron microscopy (SEM) confirmed that 0% SAPN, 35% OVA SAPN, and 100% SAPN indeed formed spherical nanocages (Figure 3i), demonstrating the successful design of modules for SAPN. To evaluate a correlation between antigen valency and nanoparticle size distribution, OVA and HrHA SAPNs with different valencies were prepared and analyzed by DLS. Nanoparticle size generally decreased as the valency of nanoparticle increased (Figure S2a,b), especially for OVA SAPN in which OVA will be more homogenously distributed due to its monomeric state. HrHA SAPNs exhibited a stronger correlation between valency and polydispersity index (PDI), with 0% and 100% HrHA SAPNs having PDIs lower than that of intermediate valency nanocages. This is likely due to the trimeric state of HrHA that cannot distribute evenly over the surface, causing populations of smaller and larger SAPNs to form, which may be higher valency and lower valency, respectively. To examine the stability of SAPNs for long-term storage, DLS was performed over time on 100% HrHA SAPN stored in phosphate buffered saline (PBS) at 4 °C. The size increased from 229 to 306 nm from day 0 to 56 and decreased to 177 nm on day 70, but the size on day 390 remained close to the initial size (257 nm) (Figure S2c). However, disassembly or degradation of the nanoparticles was not detected, indicating that the structures are robust. This observation was further supported by gel electrophoresis analysis and a number-weighted size measurement of 100% HrHA SAPNs stored in PBS at 4 for 390 days from which SAPN degradation was not detected (Figure S2d).

To assess the functional valency of SAPNs and retention of antigen recognition by antibodies, an antibody binding assay was performed on OVA-functionalized SAPNs with different valency (0–35% OVA SAPN) by ELISA with anti-OVA antibodies. According to Figure 3j, SAPNs with high valency exhibited greatly increased binding antibody levels, suggesting that the number of functional antigens on SAPNs can be tuned. This is a useful property of SAPN, as optimal antigen densities on vaccine nanoparticles have been reported to improve immune responses.^40^ To evaluate the fidelity of antigen placement, an antibody binding assay using anti-HrHA and anti-4M2e serum antibodies collected from mice with bovine serum albumin (BSA) as a negative control was conducted on 100% HrHA SAPNs. As shown in Figure 3k, both soluble HrHA-tGCN4 and HrHA presented on SAPNs were recognized by anti-HrHA antibody with similar binding levels. An anti-4M2e antibody binding assay showed significantly decreased binding antibody levels against 4M2e in 100% HrHA SAPN compared with soluble 4M2e (Figure 3). These results demonstrate that nearly all HrHA is on the outer surface of SAPNs while the majority of 4M2e antigens are not, presumably located inside the nanocage. Since there is some antibody accessibility to 4M2e antigens, we assume that either some SAPNs are not fully closed or there are some instances of 4M2e oriented differently than in the design schematic (Figure 1).

### Self-Assembled Protein Nanocages Induce Strong Humoral Immune Response against HA Antigens

**Figure 4:**
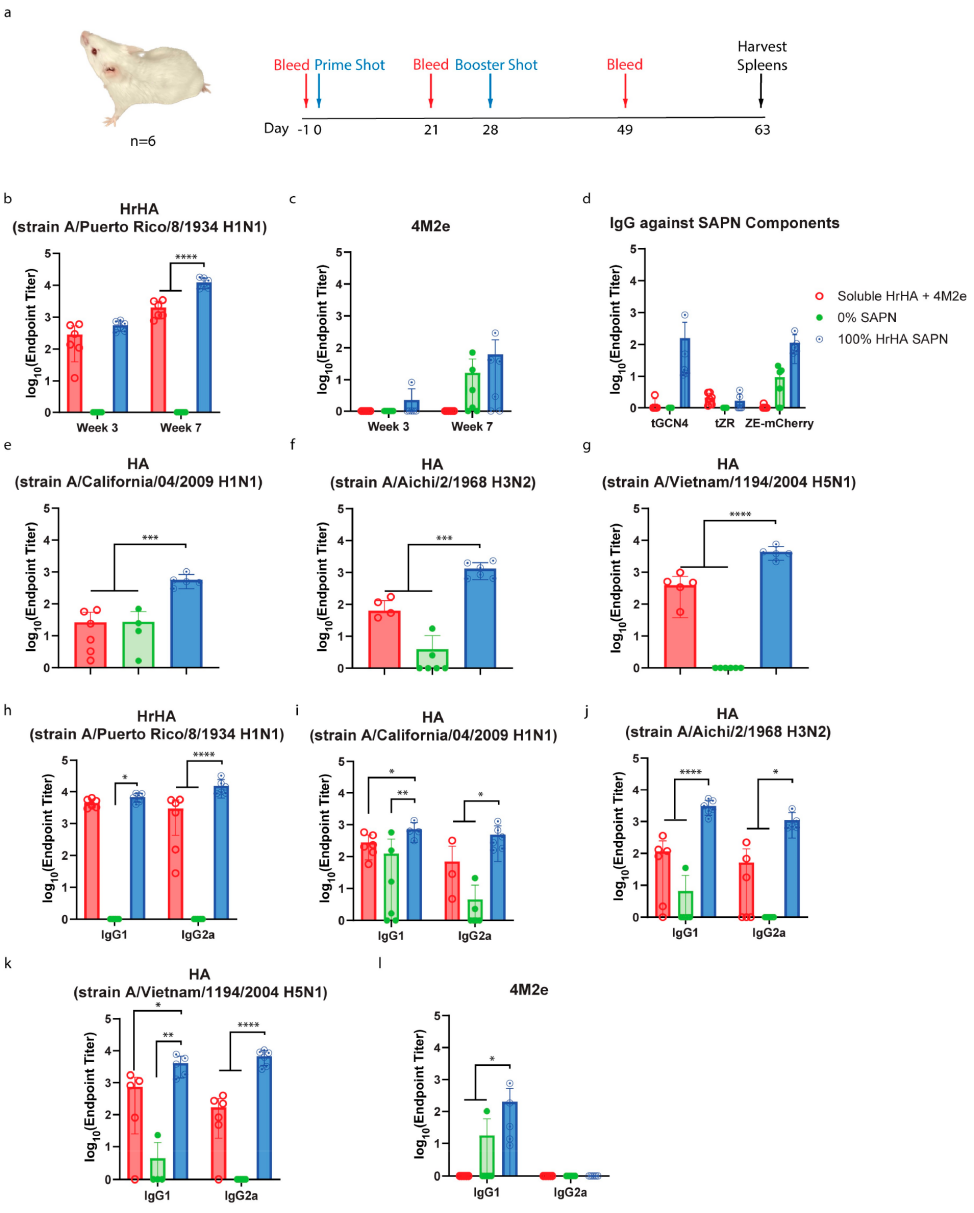
Humoral immune response of HrHA SAPNs immunized mice against antigens. (a) Schematic overview of vaccination study. IgG end point titers against soluble antigens (b) HrHA (A/Puerto Rico/8/1934 H1N1), (c) 4M2e, and (d) SAPN components including tGCN4, tZR, and ZE-mCherry of sera collected from BALB/c mice (*n* = 3 males and 3 females per group) vaccinated with soluble HrHA+4M2e, 0% SAPNs, and 100% SAPNs. IgG end point titers against soluble HA antigens derived from (e) A/California/04/2009 H1N1, (f) A/Aichi/2/1968 H3N2, and (g) A/Vietnam/1194/2004 H5N1. IgG1 and IgG2a immune responses to soluble antigens (h) HrHA, (i) H1N1 HA, (j) H3N2 HA, (k) H5N1 HA, and (l) 4M2e.

To assess the immunogenicity of HrHA SAPNs with multivalent presentation, mice were immunized and boosted intramuscularly with 100% HrHA SAPNs (8 μg dose of HrHA antigen) containing 4M2e (2 μg dose of 4M2e antigen) inside the nanocage. Their humoral immune response was compared to that of 0% SAPNs with 4M2e inside the nanocage and a mixture of HrHA-tGCN4 (8 μg dose) and tZE-4M2e (2 μg dose) (soluble HrHA+4M2e). Blood was collected at weeks 3, 7, and 11 to measure the end point titer of serum antibodies against 4M2e, HrHA, and SAPN components, tGCN4, tZR, and tZE (Figure 4a). While no significant anti-HrHA titers were seen after the prime immunization, boosting with a second dose of 100% HrHA SAPNs increased the IgG titer (week 7) against HrHA by ∼5.6-fold as compared to soluble HrHA+4M2e (Figure 4b). As expected, 0% SAPNs did not elicit a humoral response against HrHA. Sera were also collected at week 11 from mice immunized with 100% HrHA SAPNs to assess the durability of the humoral immune response against HA stalk. IgG titers were higher than those collected on week 7 by ∼10-fold (Figure S4), demonstrating that the humoral immune response continued to increase after the 100% HrHA SAPN booster for 7 weeks without a third boost. Contrary to high anti-HrHA titers, the 100% HrHA SAPN immunized group showed no difference in antibody titer against 4M2e as compared to control mice vaccinated with 0% SAPNs and soluble HrHA+4M2e (Figure 4c). In alignment with the anti-4M2e antibody binding assay (Figure 3), this indicates that most 4M2e antigens inside the nanocages are inaccessible by B cell receptors, confirming the fidelity of antigen placement in the nanocage design. Contrary to our nanocage design, it was demonstrated that, when M2e antigens were presented on external surfaces of nanocages such as human heavy chain ferritin (rHF)^41^ or encapsulin,^42^ the nanocages could promote potent humoral immune responses against M2e. We also assessed the off-target immune response against SAPN scaffolds by performing ELISA using tGCN4, tZR, and tZE fused to mCherry (ZE-mCherry) as binding targets. As indicated in Figure 4d, no significant humoral immune responses against any of the components were seen in SAPN immunized mice. This also suggests that the anti-His-tag humoral immune response was not induced, as His-tagged mCherry was not recognized by the serum antibodies. Given that His-tags were fused to the C-termini of HrHA-tGCN4 and tZE-4M2e (Table S1), His-tags located inside the nanocage could have been shielded by SAPN scaffold components from being recognized by B cells. Therefore, the result demonstrates that SAPN is a vaccine platform that can prevent irrelevant immune responses despite its viral mimicking geometry. This could be especially useful for weak antigens that cannot be matched with natural VLP or *in silico* designed icosahedral nanocage coat proteins.^43^

Next, we evaluated the breadth of the antibody cross-reactivity. HA antigens derived from strains A/California/04/2009 H1N1, A/Aichi/2/1968 H3N2, and A/Vietnam/1194/2004 H5N1 were selected to reflect major phylogenetic HA clades and the subtypes of currently circulating dominant strains (H1N1 and H3N2).^44^ 100% HrHA SAPNs substantially improved IgG titers (week 7) against HA antigens from H1N1, H3N2, and H5N1 by 22-, 34-, and 13-fold, respectively, compared to soluble HrHA+4M2e (Figure 4e–g). Importantly, although HA from phylogenetically distant H3N2 contains only a small portion of residues overlapping with HrHA (Figure S3), an enhanced humoral immune response was induced by 100% HrHA SAPNs, likely due to the multivalent presentation and controlled orientation of antigens. Thus, 100% HrHA SAPN immunized mice acquired a broad antibody repertoire. Taken together, these results demonstrate potent humoral immune responses against different HA subtypes, including phylogenetically distant subtypes, promoted by 100% HrHA SAPNs. In contrast, universal influenza vaccines prepared by coating desolvated 4M2e nanoparticles with the same HrHA antigens (Strain A/Puerto Rico/8/1934) did not elicit detectable humoral immune response against phylogenetically distant H3N2 HA.^5^ This highlights the significance of valency and the controlled orientation of antigens for strong, broad humoral immunity.

To study IgG responses in depth, titers of IgG isotypes were also measured. 100% HrHA SAPNs enhanced HrHA-specific IgG2a by 5-fold while the IgG1 response was comparable to that elicited by soluble HrHA+4M2e (Figure 4h). At 11 weeks, anti-HrHA IgG2a titers, but not IgG1 titers, were significantly higher than those at 7 weeks, demonstrating persistent isotype trends (Figure S4). Titers of cross-reactive IgG isotypes, especially IgG2a, were significantly augmented by 100% HrHA SAPNs. 100% HrHA SAPNs improved IgG1 and IgG2a titers by ∼2.6- and ∼7-fold against H1N1 HA, ∼27- and ∼21.7-fold against H3N2 HA, and ∼5.5- and ∼39-fold against H5N1 HA, respectively (Figure 4i–k). IgG2a response is critical for antiviral activity, as this isotype engages Fc receptors of effector cells and promotes effector functions such as antibody-dependent cellular cytotoxicity.^12,45^ Therefore, the enhanced IgG2a immune response is a promising outcome to fight influenza. Although a very low anti-4M2e IgG1 titer was detected in mice administered with 100% SAPNs, since total IgG was not detected for 100% SAPNs and no IgG, total or IgG1, was detected for 0% SAPNs, we can conclude that the bulk of SAPN maintained their structure in vivo and matched the design with internal 4M2e (Figure 4).

### Self-Assembled Protein Nanocages Enhance Dendritic Cell Activation and T Cell Response against HA

**Figure 5:**
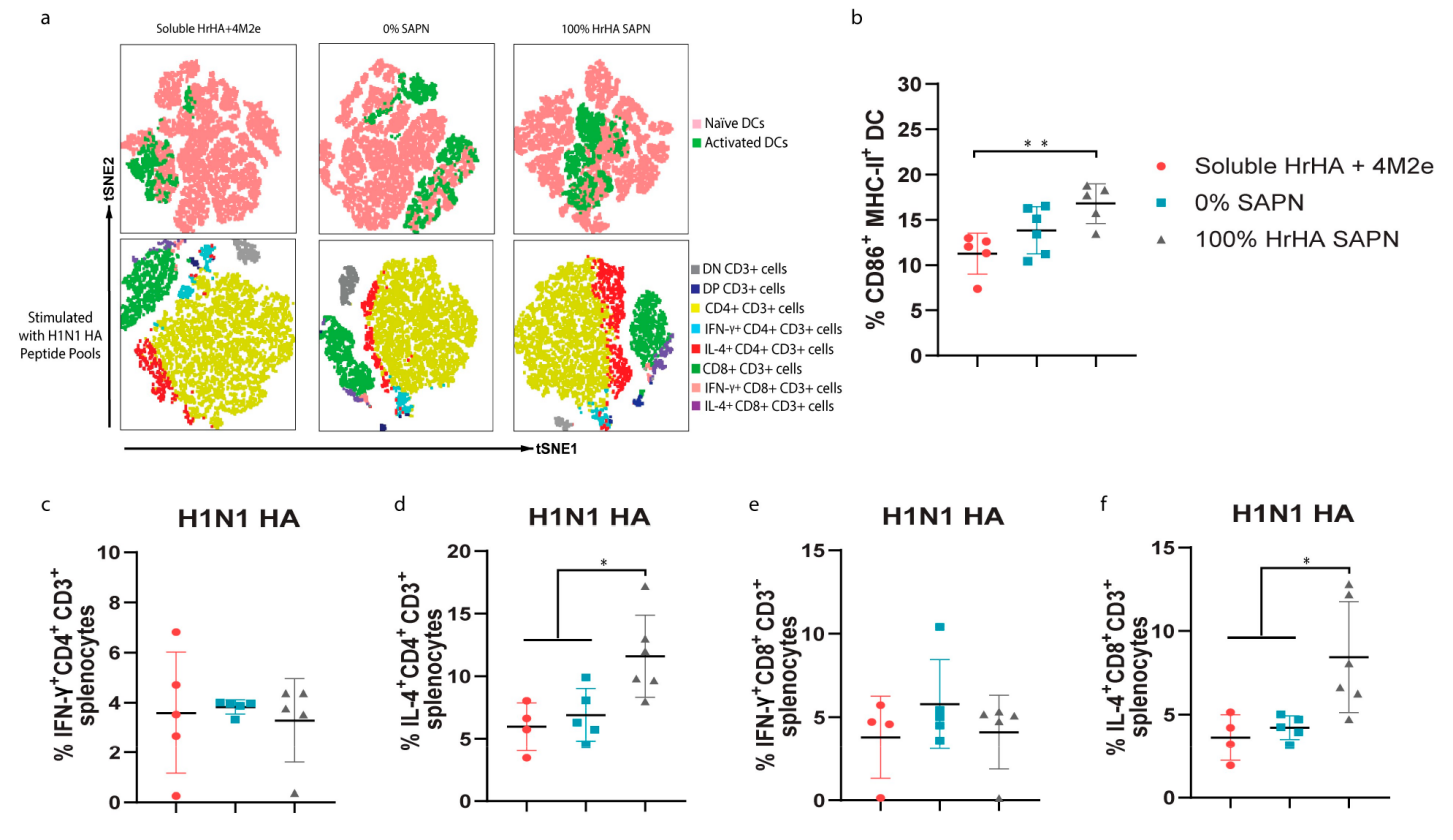
Cellular immune response of HrHA SAPNs immunized mice. (a) Distribution of DCs and T cell subsets from vaccinated mice analyzed by surface markers and intracellular cytokine staining and visualized by t-distributed stochastic neighbor embedding (tSNE). (b) Percentage of CD86^+^ MHC-II^+^ CD11c^+^ DCs. Percentages of (c) IFN-γ^+^ CD4^+^ CD3^+^, (d) IL-4^+^ CD4^+^ CD3^+^, (e) IFN-γ^+^ CD8^+^ CD3^+^, and (f) IL-4^+^ CD8^+^ CD3^+^ T cells after restimulation by H1N1 HA peptide pools (Strain A/California/04/2009). The stained splenocytes were harvested from mice vaccinated with soluble HrHA+4M2e (red), 0% SAPNs (cyan), and 100% HrHA SAPNs (gray).

The cellular immune response, including cytotoxic CD8^+^ T cells and helper CD4^+^ T cells, plays a critical role in eradicating pathogens and orchestrating the activation of B and T cells. It was, therefore, of interest to explore the distribution of activated T cell subsets. First, to understand the effect of SAPNs on the crosstalk between antigen presenting cells and the activation of T cells, splenocytes harvested from immunized mice were stained and gated to identify dendritic cells (DCs, CD11b+), the most efficient antigen-presenting cells (Figure S5). Notably, when mice were immunized with 100% HrHA SAPNs, a higher percent population of activated DCs (CD86+, MHCII+), professional antigen presenting cells bridging innate and adaptive immunity, were observed as compared to the soluble HrHA+4M2e group (Figure 5a,b). This suggests that 100% HrHA SAPNs were effectively internalized by dendritic cells *in vivo*. However, 0% SAPN did not significantly enhance the activation of DCs above soluble antigens, indicating that 4M2e antigens inside the caged nanostructure alone were not sufficient for strong activation of DCs.

In parallel, antigen-specific T cells from splenocytes were identified by restimulating them with H1N1 (A/California/04/2009) HA peptide pools, staining, and gating based on T cell surface markers and intracellular cytokines (Figure 5a). There was no evident difference in the activation of IFN-γ^+^ CD4^+^ T cells (Th1) between groups administered with soluble HrHA+4M2e, 0% SAPNs, and 100% HrHA SAPNs (Figure 5a,c). However, 100% HrHA SAPNs significantly increased the percent population of IL-4^+^ CD4^+^ T cells (Th2) from H1N1 HA peptide stimulated T cells as compared to soluble HrHA+4M2e and 0% SAPNs (Figure 5a,d). Analogous to the increased population of H1N1 HA specific IL-4^+^ CD4^+^ T cells by 100% HrHA SAPNs, the percent population of H1N1 HA specific IL-4^+^ CD8^+^ T cells from 100% HrHA SAPN immunized mice was higher than that from soluble HrHA+4M2e and 0% SAPN immunized mice. However, the percent population of IFN-γ^+^ CD8^+^ T cells from H1N1 HA stimulated T cells remained the same among the different groups. HrHA, which has >90% of sequence overlapped with A/California/04/2009 H1N1 HA,^5^ could consist of more Th2 epitopes than Th1 epitopes. B cells can also promote the development of Th2 cells that provide help in B cell activation by secreting growth factors such as IL-4.^12,46,47^ Strong B cell activation by the multivalent presentation of HrHA could have promoted Th2 development. However, the antibody titers indicated IgG2a (Th1) titers greater than or similar to those of IgG1 (Th2) for both H1N1 strains (Figure 4h,i). In any case, the overall population of activated T cells established in mice vaccinated with 100% HrHA SAPNs was generally improved compared to soluble HrHA+4M2e and 0% SAPNs. Taken altogether, the humoral and cellular HA specific responses suggest that there is a balanced immune response. A recent study using HA/CpG nanoparticles formed by polycationic polyethylenimine reported that the vaccine balanced Th1/Th2 responses and enhanced cross protection against influenza.^48^ Th1 is associated with cellular immune response of CD8^+^ T cells, while Th2 is known for promoting antibody production and can, thus, decrease extracellular viral loads.^49−51^ Therefore, balanced Th1/Th2 responses can be beneficial for the effective clearance of influenza.

In contrast to H1N1 HA, restimulation with 4M2e resulted in weak Th2 responses but a high percent population of IFN-γ^+^ CD8^+^ T cells (Figure S6). However, all groups showed similar populations of 4M2e-specific T cells despite the enhanced activation of DCs observed in 100% HrHA SAPN vaccinated mice. This may be possibly due to the restimulation of T cells with full 4M2e protein rather than M2e peptide pools, resulting in relatively slow antigen processing and loading onto MHC molecules for T cell reactivation.^52,53^ Different studies have also reported inconsistent results on T cell responses induced by M2e due, in part, to discrepancies in vaccine platforms, immunization protocols, etc.^9^ Although the immune defense mechanism induced by M2e remains incompletely defined, effective protection conferred by humoral immune responses to M2e has been demonstrated.^5,9,54^ This implies that placement of 4M2e on external surfaces of SAPNs can be an effective strategy for the formulation of a universal influenza vaccine in the future. In this work, 4M2e was placed inside the nanocages instead of outside in order to examine spatial control of antigens. We also note that the IFN-γ dominant T cell responses elicited by 4M2e can be complemented by enhanced IL-4 Th2 immune responses induced by HrHA SAPNs, demonstrating the advantages of using multiple antigens.

## Conclusion

Antigenic variation has been regarded as a hallmark of escape from acquired immune responses. When a population is infected by influenza viruses, the virus mutants that are not recognized and eliminated by the host immune responses can replicate and evolve under multiple rounds of selection pressure.^55^ The antigenic variation underlying the failure of vaccines can occur by mutating small portions of the virus or forming heterosubtypic virus strains, known as antigenic drift and shift, respectively.^56−59^ Hence, the state of the art currently requires an annual update of flu vaccines against likely seasonally circulating viruses. Yet, the seasonal influenza vaccines have not demonstrated high effectiveness against viral mutants due to a high frequency of antigenic variation,^3,4^ making it not cost-effective and even leading to a lack of preparedness for an outbreak of unexpected pandemic virus. Thus, the outbreak of Influenza A strains and their immune escape via antigenic variation highlight the need for universal flu vaccines requiring the use of highly conserved regions of the virus as subunits.

Highly conserved HA stalk and M2e are considered desirable targets for the development of universal influenza vaccines.^5−11^ Nevertheless, the standalone immunosubdominant antigens often show poor immunogenicity compared with the dominant variable antigens, such as HA head domains. Although they can be conjugated to nanoparticles to render them immunogenic, there remain some challenges to optimize the properties of nanoparticles for enhanced immune responses because the efficacy of subunit vaccines largely depends on physicochemical properties, namely size, valency, and stability.^14,60−63^ This work outlines the design strategy for SAPN by engineering trimeric GCN4 and heterodimeric leucine zippers and its potential as a modular scaffold for the development of a universal flu vaccine. The platform exhibited improved humoral immune responses directed to HA by presenting multiple copies of trimeric conformational HA stalks in a highly repetitive array, while minimizing off-target responses. The significance of trimeric HA stalk conformation and multivalency was also demonstrated by H1-based stabilized stem nanoparticles developed by Yassine et al.^31^ In the study, ferritin nanoparticles with multivalent presentation of trimeric HA stalks on the surface elicited broadly cross-reactive humoral immunity and completely protected mice against a lethal heterosubtypic H5N1 influenza challenge. Yet, ferritin nanoparticles can induce antiparticle humoral immune responses, depending on the species.^64,65^ Therefore, an advantage of SAPN as a vaccine platform is minimizing off-target immune response against scaffolds such as VLPs, ferritins, or designed assemblies^66−70^ while maximizing immune responses against influenza antigens. By redirecting immune responses to the highly conserved domains, SAPNs may address limitations of vaccines associated with original antigenic sin, which favors immune responses against variable immunodominant domains of influenza antigens such as HA head; repeated exposure to influenza has established immune memory that strongly reacts to the immunodominant head overriding humoral immune response against HA stalk.^71^ We and others have reported that anti-M2e humoral immune responses are critical for protection against influenza viruses.^5,54^ However, we chose the design of SAPNs to incorporate 4M2e antigens inside the nanocage to test the spatial control of SAPNs, as our previous coated nanoparticle designs were not able to spatially segregate the two antigens. Self-assembling nanoparticles such as stabilized HA stem ferritin nanoparticles^31^ also have hollow caged structures, but they cannot easily incorporate multiple copies of antigens inside the structure due to their small interior cavity diameter (∼7–8 nm).^72,73^ Furthermore, many nanocages consist of identical subunits, including ferritin with 24, rendering them difficult to recombinantly present different antigens in a controlled manner. SAPN thus offers the advantage of spatially controlling antigen presentation with the capacity to incorporate different antigens due to the use of three different building blocks. These features also make SAPN a useful tool for the immunological evaluation of different antigens. For example, the importance of the anti-M2e humoral immune response can be assessed by comparing SAPNs internally and externally functionalized with M2e. With this proof of concept, future designs will enable anti-M2e humoral immune responses for broad protection against influenza by functionalizing SAPNs with both 4M2e and HrHA on the external surface.

While HA stalks and M2e are attractive targets for cross-reactive immune responses and have conferred protection against challenge by heterosubtypic influenza strains in mice,^5^ sole exploitation of these antigens may not be sufficient for larger animals and humans. HA stalks generally induce inferior neutralizing humoral immunity compared to HA head,^74,75^ and HA stalks may even cause antibody-dependent enhancement (ADE) of influenza infection by enhancing viral fusion via destabilization of HA stalks.^76^ The mechanism of ADE-mediated influenza infection was examined using a few selected antibodies targeting H3N2 stalks, and therefore, there remains uncertainty over whether other anti-HA stalk antibodies also result in ADE of infection. Hence, the design of broadly cross-reactive influenza vaccines would be facilitated by screening anti-HA stalk antibodies that are capable of neutralizing divergent virus strains and identifying the epitopes recognized by the antibodies.^77,78^ Other studies showed the importance of Fc-FcγR interactions on effector cells for broadly neutralizing activity of anti-HA stalk and HA head antibodies.^74,79^ One suggests that engagement of effector cells, mediated especially by IgG2a isotype, plays a critical role in neutralizing activities of anti-HA stalk antibodies.^79^ SAPNs significantly improved IgG2a titers against stalks of different HA subtypes, implying that the nanocage may improve the neutralizing activities of anti-HA stalk antibodies, which could be tested in future work. Many influenza vaccines have also utilized M2e, but the correlates of protection remain undefined or limited.^9^ Furthermore, M2e exhibits weaker immunogenicity than HA. It is therefore recommended to use M2e antigens together with other antigens.^5,9,80,81^ Of note, recent findings highlight the significance of T cell responses for broadly cross-reactive immunity against influenza.^82−85^ Broadly cross-reactive T cell responses were shown to be potent and significantly contribute to broad protection against influenza infections which was independently of hemagglutination inhibition (HAI) titer, a major indicator of neutralizing HA-specific humoral immunity.^85^

Therefore, to further enhance potency of universal influenza vaccine, incorporation of strong T cell epitopes from viral polymerase subunit (PB1) and/or nucleoprotein (NP) inside nanocages can be designed in concert with the HA stalk and M2e. Finally, by demonstrating the formation of SAPNs decorated with OVA, we envision that the nanocage could be harnessed as a “plug-and-display” modular vaccine with the choice for external or internal antigen presentation as appropriate. Altogether, SAPN demonstrates a combination of features that may not be easily attainable with existing vaccine platforms.

## Methods

### Molecular Dynamics Simulation

HrHA-tGCN4 protein model was prepared using Chimera and was compared to the structure predicted by ColabFold for the validation of *ab initio* protein structure assembly. Following the initial protein structure prediction, each HrHA-tGCN4 monomer was fused to a tZE coiled coil via the formation of a disulfide bond. The assembled trimeric HrHA-tGCN4/tZE protein containing three units of tGCN4/tZE was simulated by GROMACS with the Amber99SB-ildn force field. The protein was solvated with water molecules using the TIP3 model and refined by performing energy minimization. Before the energy minimization, sodium and chloride ions replaced random water molecules to neutralize the overall charge of the system. The simple steepest descent minimization was performed with the Particle Mesh Ewald method for a maximum of 50000 steps. Then, the solvated system was stabilized by equilibration in constant number, volume, temperature (NVT) and constant number, pressure, temperature (NPT) ensembles at 298 K as previously outlined by Park et al.^14^ Posre and LINCS were utilized to apply positional restraints on the heavy atoms of HrHA-tGCN4 and hydrogen bonds, respectively. After the NVT and NPT equilibration, the molecular dynamics were simulated for 500 ps in the NPT ensemble using the leapfrog algorithm. The simulation was then visualized by VMD and Chimera.

### Vector Constructs and Synthesis of Recombinant Proteins

The gene sequences encoding His-tagged HrHA-tGCN4 and H1N1 (A/California/04/2009) HA recombinant proteins were cloned into vector pcDNA3.1 with codon optimization (Gene Universal). All gene and protein sequences are given in Table S1. HrHA-tGCN4 and H1N1 HA were expressed via transient transfection of mammalian Expi293F cells by using the Expi293 Expression System kit (Thermo Fisher Scientific) as per the manufacturer’s instructions. In brief, Expi293F cells were cultured in Expi293 Expression Medium (Thermo Fisher Scientific) in polycarbonated vented Erlenmeyer flasks (Thermo Fisher Scientific) at 37 °C, 8% CO_2_ on a shaker at 125 rpm and transfected with complexes formed from HrHA-tGCN4 or H1N1 HA encoding pcDNA3.1 and ExpiFectamine 293 Reagent diluted in Opti-MEM I Reduced Serum Medium (Thermo Fisher Scientific). After 18–20 h of incubation, Transfection Enhancer was added to the transfected Expi293F cells. The cells were then harvested 5 days post-transfection and lysed by sonication on ice in a lysis buffer containing 20 mM imidazole, 300 mM NaCl, and 50 mM NaH_2_PO_4_. The His-tagged HrHA-tGCN4 and H1N1 HA proteins were boundto Ni-NTA agarose and eluted with 300 mM imidazole buffer after washing with 50 mM imidazole buffer.

The tZE-4M2e expression construct was synthesized by cloning tZE-4M2e into pETDuet-1 and pET17 with codon optimization (GenScript). Protein was expressed in *E. coli* BL21 Star (DE3). The cells were precultured in 10 mL of LB medium with 100 μg/mL ampicillin for 16 h at 37 °C on a shaker at 200 rpm. The preculture was diluted into 1 L of LB media containing 100 μg/mL ampicillin, and when the cell density reached OD600 0.4–0.6, protein expression was induced by adding isopropyl-β-d-1-thiogalactopyranoside to a final concentration of 1 mM and incubated at 37 °C with shaking at 120 rpm for 5 h. The cells were sonicated on ice in a lysis buffer containing 8 M urea, 10 mM Tris-Cl, and 100 mM NaH_2_PO_4_ with pH 8.0. The tZE-4M2e proteins were eluted with 8 M urea buffers with pH 5.9 and 4.5 from a column packed with Ni-NTA resin after washing with 8 M urea buffer with pH 6.3.

Following purification, the buffers of HrHA-tGCN4, H1N1 HA, and tZE-4M2e recombinant proteins were exchanged with phosphate buffered saline (PBS) using 3 kDa molecular weight cutoff Amicon Ultra-4 Centrifugal Filter Unit (Millipore Sigma). Endotoxin was removed from recombinant proteins by using Pierce High-Capacity Endotoxin Removal Spin Columns (Thermo Fisher Scientific) as per the manufacturer’s instructions. Endotoxin levels were measured by ToxinSensor Chromogenic LAL Endotoxin Assay Kit (GenScript) to verify that the levels were maintained below the endotoxin limit of 15 EU/mg.^86^ Lyophilized tGCN4 and tZR peptides were obtained by solid-phase peptide synthesis (GenScript).

### Analysis of Protein Expression and Structure

Proteins were identified by SDS-PAGE and Western blot. Soluble HrHA-tGCN4, OVA-tGCN4, and ZE-4M2e were mixed with a Laemmeli buffer solution (Biorad) containing dithiothreitol (DTT) and incubated at 95 °C for 5 min. For the analysis of quaternary protein structure, soluble HrHA-tGCN4 and OVA-tGCN4 were stabilized by 2% and 3% glutaraldehyde cross-linkers and incubated with Laemmeli buffer without DTT at 95 °C for 5 min. After the samples were cooled, they were eluted through 12% SDS-PAGE gel for 80 min at 150 V in tris-glycine SDS gel electrophoresis buffer. The separated proteins were then stained with Coomassie Blue R-250. A second, unstained gel was used to transfer proteins to a Western blot membrane. The membrane was blocked with PBS containing 5% (w/v) dry milk and 0.1% Tween-20 and incubated with Penta-His Alexa Fluor 488 Conjugate (Qiagen) to analyze His-tagged HrHA-tGCN4, OVA-tGCN4, and tZE-4M2e. For the analysis of heterodimerization of tZR and tZE-4M2e, 5 μL of 200 μg/mL tZR, 200 μg/mL of tZE-4M2e, or 400 μg/mL of an equimolar mixture of tZR and tZE-4M2e was mixed with 5 μL of Nu-PAGE lithium dodecyl sulfate loading buffer (Invitrogen) and incubated at 95 °C for 5 min. Protein samples were eluted through 4–12% Bis-Tris gel (Invitrogel) for 50 min at 120 V, and the gel was stained with Imperial Protein Stain (ThermoFisher Scientific). The gel was destained with DI water overnight. All of the stained gels and membranes were imaged by a Gel Doc X + Gel Documentation System (Biorad).

A Malvern OmniSEC integrated system (Malvern Panalytical) with a SRT SEC-300 analytical SEC column (Sepax) was used to assess structures of HrHA-tGCN4 and OVA-tGCN4 as previously described.^87^ A bovine serum albumin standard was used to perform calibration, and the chromatogram profiles were assessed by a refractive index, right angle light scattering, and viscometer. A refractive index increment value (d*n*/d*c*) of 0.185 was provisioned into the software to calculate the molecular weight of the peaks, and each peak area was quantified at 254 nm wavelength to determine percent fractions of sample.

CD was performed with a ChiraScan-plus CD spectrometer (Applied Photophysics) in the wavelength range from 200 to 260 nm to analyze the secondary structures of tGCN4, tZR, tZE-4M2e, OVA-tGCN4, and HrHA-tGCN4. Each protein was diluted to 0.5 mg/mL with PBS in a 0.5 mm one-piece stoppered quartz cuvette (Applied Photophysics), and CD signals were measured as previously outlined by Park et al.^14^

### SAPN Fabrication

Prior to SAPN assembly, tGCN4 protein in PBS was treated with 10 mol equiv of aldrithiol-2 (MilliporeSigma) in methanol for 1 h at 25 °C to inhibit disulfide bond formation between tGCN4 coiled coils. For example, 500 μg of HrHA-tGCN4 (15.2 nmol) in 1 mL of PBS was reacted with 33 μg of aldrithiol-2 (152 nmol) in 100 μL of methanol. OVA-tGCN4 or HrHA-tGCN4 treated with aldrithiol-2 was exchanged into PBS by a 3 kDa molecular weight cutoff Amicon Ultra-4 Centrifugal Filter Unit. Alridthiol-2 treated tGCN4 peptides were placed in a sealed Spectra Por membrane tube (Cole Palmer) with 0.1–0.5 kDa molecular weight cutoff for dialysis in PBS to remove any unreacted aldrithiol-2 molecules and methanol. To generate SAPN building blocks, tGCN4 (fusion proteins or peptide, depending on antigens desired) was mixed with tZR or tZE-4M2e in equimolar concentration, and prior to mixing, tZE-4M2e was reduced by 10 mM DTT for 1 h at 25 °C to prevent disulfide bond formation between tZE-4M2e proteins and exchanged into PBS by a 3 kDa molecular weight cutoff Amicon Ultra-4 Centrifugal Filter Unit. To be more specific for 100% SAPN building blocks, 200 μL of 0.8225 μg/μL HrHA-tGCN4 (25 μM) was mixed with 200 μL of 0.4245 μg/μL reduced tZE-4M2e (25 μM) or 200 μL of 0.0705 μg/μL tZR (25 μM) to yield 25 μM HrHA-tGCN4/tZE-4M2e and 25 μM HrHA-tGCN4/tZR. Similarly for 0% SAPN building blocks, tGCN4/tZE-4M2e and tGCN4/tZR at a final concentration of 25 μM were generated by mixing tGCN4 with tZE-4M2e and tZR. SAPNs were assembled by mixing the SAPN blocks. For example, to generate 33% HrHA SAPNs, a tZR mixture was prepared by mixing and incubating 66 μL of HrHA-tGCN4/tZR with 134 μL of tGCN4/tZR for 1 h at 25 °C. At the same time, 66 μL of HrHA-tGCN4/tZE-4M2e and 134 μL of tGCN4/tZE-4M2e were mixed to produce a tZE-4M2e mixture. After the incubation, the tZR mixture was added to the tZE-4M2e mixture and incubated for 4 h at 25 °C to yield 400 μL of 33% SAPNs at a final concentration of 25 μM. For the formation of 100% HrHA SAPNs, 200 μL of HrHA-tGCN4/tZR and 200 μL of HrHA-tGCN4/tZE-4M2e were mixed and incubated for 4 h at 25 °C.

### SAPN Characterization

The hydrodynamic size distribution of SAPNs was assessed by dynamic light scattering (DLS) with a Malvern Zetasizer Nano ZS instrument. Three measurements were taken per sample at a scattering angle of 173° with a beam wavelength of 633 nm. A refractive index of 1.45 was used to assess both OVA and HrHA SAPNs, while a viscosity of 0.8882 cP with a refractive index of 1.33 was used for PBS. The concentrations of SAPNs and soluble proteins were measured with a Pierce bicinchoninic acid (BCA) protein assay according to the manufacturer’s instructions (Thermo Fisher Scientific).

The morphology of SAPNs was evaluated by TEM. Five μL of SAPNs was placed on a 300-mesh carbon film supported copper grid (MilliporeSigma) for 10 min and washed by dipping in 5 μL of deionized water for 30 s. The excess water was wicked off with a Kimwipe. The sample was stained with 5 μL of 1% phosphotungstic acid solution for 10–15 s. After negative staining, the grid was washed with 5 μL of deionized water, immediately wicked off, and dried overnight. The TEM samples were imaged at 100 kV using a JEOL 100 CX-II TEM instrument. A high-resolution TEM image of 100% HrHA SAPN was taken by Dr. Srihari Nagendra Ravi Kiran Koripella and Dr. Ricardo Guerrero-Ferreira in the Robert P. Apkarian Integrated Electron Microscopy Core at Emory University.

For SEM imaging, 5 μL of SAPNs in 0.9% NaCl buffer was dropped on a silicon chip attached to an aluminum SEM stub and allowed to dry overnight. The mounted sample was sputter-coated with platinum and visualized at 5–15 kV with a Hitachi SU8010 SEM.

### Antibody Binding Assay for OVA and HrHA SAPN

OVA SAPNs and HrHA SAPNs with 10-fold serial dilutions (10^–4^–1 ng/μL and 10^–6^–1 ng/μL, respectively) were coated onto Maxisorp 96 well immune assay plates (Nunc) and incubated overnight at 25 °C. The next day, the coated plates were washed three times with PBS containing 0.1% Tween-20 and each well was blocked with 1% bovine serum albumin (BSA) in PBS for 1 h at 25 °C. Then, the plates were washed three times and incubated with HRP-conjugated rabbit anti-OVA antibodies (Thermo Fisher Scientific) diluted at 1:3,000 for 1 h at 25 °C. For anti-HrHA and anti-4M2e antibody binding assay, the plates were incubated with anti-HrHA and anti-4M2e serum antibodies, which were harvested from mice administered with 2 doses of 20 μg of HrHA-tGCN4 and 20 μg of tZE-4M2e, diluted at 1:5000 and 1:2500, respectively, for 1 h at 25 °C. Afterward, plates incubated with the anti-HrHA and anti-4M2e serum antibodies were washed and incubated with a 1:5000 dilution of HRP-conjugated goat antimouse IgG (H+L) (SouthernBiotech) for 1 h at 25 °C. Each well was washed three times and revealed with 1-Step Ultra TMB substrate solution (Thermo Fisher Scientific) for 20 min. The enzymatic activity of HRP was stopped by adding 2 N H_2_SO_4_ solution (Thermo Fisher Scientific). Absorbance at 450 nm was measured on a BioTek Synergy H4 Micro plate reader with the correction wavelength set to 540 nm.

### Animal Immunization

In this study, six mice (BALB/C strain, 6–8 weeks of age, 3 male, 3 female) were intramuscularly immunized with a soluble mixture of 8 μg of HrHA-tGCN4 and 2 μg of tZE-4M2e, 10 μg of 0% SAPNs, or 10 μg of 100% HrHA SAPNs (containing 8 μg of HrHA-tGCN4 and 2 μg of tZE-4M2e) in the thigh muscles of the hind limb. Prior to vaccination, all animals were acclimatized for at least 5 days. An identical injection was given 4 weeks post prime. Blood sera were collected from the jugular vein of mice with anesthesia 3, 7, and 11 weeks after prime. On week 8, mice were euthanized by CO_2_ asphyxiation to harvest spleens. All animal experiments were performed in accordance with guidelines and regulations approved by the Georgia Institute of Technology Institutional Animal Care and Use Committee (IACUC) under approved protocol A100576.

### Enzyme-Linked Immunosorbent Assay for Antibody Titers

The titers of serum IgG, IgG1, and IgG2a were measured using an enzyme-linked immunosorbent assay (ELISA). Briefly, 1 μg/mL of each flu antigen, including H1N1 HrHA (derived from A/Puerto Rico/8/1934), H1N1 HA (A/California/04/2009), H3N2 HA (A/Aichi/2/1968) (SinoBiological), H5N1 HA (A/Vietnam/1194/2004) (SinoBiological), and tZE-4M2e, in PBS was coated onto Maxisorp 96 well immune assay plates during overnight incubation at 25 °C. For the evaluation of off-target immune responses, Maxisorp 96-well plates were coated with 1 μg/mL of tGCN4, tZR, or ZE-mCherry. The next day, each well was washed three times with PBS containing 0.1% Tween-20 and blocked with 1% BSA-supplemented PBS for 2 h at 25 °C. Each well was incubated with serially diluted sera for 1 h at 25 °C and washed three times. Then, a 1:5000 dilution of HRP-conjugated goat antimouse IgG (H+L) (SouthernBiotech), IgG1 (SouthernBiotech), or IgG2a (SouthernBiotech) secondary antibodies was added to each well, incubated for 1 h at 25 °C, and washed three times. Each well was developed with 1-Step Ultra TMB substrate solution for 15 min and stopped with 2 N H_2_SO_4_ solution. Absorbance values at 450 nm were measured on a BioTek Synergy H4 Micro plate reader with the correction wavelength set to 540 nm.

### Splenocyte Characterization

Surface and intracellular cytokine staining assays were conducted after stimulation of splenocytes with antigens as described previously.^14^ In brief, the spleens were harvested, triturated gently, and strained through 70 μm strainers. Splenocytes were then resuspended in complete RPMI 1640 medium supplemented with HEPES, l-glutamine (Thermo Fisher Scientific), and 10% FBS and incubated with ACK lysing buffer (Thermo Fisher Scientific) at 25 °C for 9 min to remove red blood cells. In each well of round-bottom 96 well plates, 1 × 10^6^ splenocytes were seeded and stimulated with 2 μL of reconstituted PepTivator Influenza A (H1N1) HA stock solution (Miltenyi Biotec) or 10 μg/mL of full length tZE-4M2e protein. For mock restimulation, 0.5 μL of 2 μg/mL phorbol 12-myristate 13-acetate and 0.5 μL of 100 μg/mL calcium ionophore were added to each well. After 3 h incubation at 37 °C, 5% CO_2_, a 2 μL mixture of 50x brefeldin A and 50x monensin (BioLegend) was added to each well to block cytokine secretion during cell activation for an additional 3 h. T cells were stained with Zombie Violet fixable viability dye, PerCP anti-CD3, FITC anti-CD8, and APC/Cy7 anti-CD4 (BioLegend) prior to intracellular cytokine staining. After surface staining, restimulated T cells were fixed with 3.7% formaldehyde and stained with PE anti-IFN-γ and PE/Cy7 anti-IL-4 (BioLegend) in permeabilization buffer (Thermo Fisher Scientific). For staining dendritic cells, cells without-restimulation were surface-labeled with Zombie Violet fixable viability dye, APC/Cy7 anti-CD11c, PE anti-CD86 (BioLegend), and FITC anti-MHC II (Thermo Fisher Scientific). All cells were resuspended in 1% BSA-supplemented PBS and analyzed by Cytek Aurora flow cytometry (Cytek Biosciences). Data analysis was conducted using FlowJo software. The gating strategy is presented in Figure S5. For visualizing a population distribution of gated splenocytes, t-distributed stochastic neighbor embedding (t-SNE) was implemented using the Barnes–Hut algorithm via FlowJo software.

### Statistical Analysis

All statistical analyses were performed with GraphPad Prism 9. One-way ANOVA with Turkey’s posthoc multiple comparison analysis was used to calculate *p* values for statistical comparison. Statistical significance was determined as follows: (*) for *p* ≤ 0.05, (**) for *p* ≤ 0.01, (***) for *p* ≤ 0.001, and (****) for *p* ≤ 0.0001. All data plotted with error bars are reported as means with a standard deviation.

## Supporting Information Available

Circular dichroism and predicted structures of recombinant proteins, DLS data of OVA- and HrHA-functionalized SAPNs, sequence alignment of different HA subtypes, end point titers of anti-HrHA IgG, IgG2a, and IgG1 collected on week 11 from HrHA SAPN immunized mice, gating strategy to identify activated DCs and T cell subsets after surface and ICS staining, 4M2e-responsive T cells from HrHA SAPNs immunized mice, and a list of recombinant protein sequences (PDF)
Movie S1. MD simulation of HrHA-tGCN4/tZE and tGCN4/tZR folding and interactions (MP4)
Movie S2. MD simulation of electrostatic repulsion between SAPN coiled coils (MP4)
Movie S3. MD simulation of tGCN4 trimerization (MP4)
