# Supplementary material for: Development of Self-Assembled Protein Nanocage Spatially Functionalized with HA Stalk as a Broadly Cross-Reactive Influenza Vaccine Platform
Source: ACS Nano. 2023 Dec 12;17(24):25045–60. doi: 10.1021/acsnano.3c07669 (PMC10753887; doi:10.1021/acsnano.3c07669)
Supplement: Supplementary file 1 — nn3c07669_si_001.pdf [file nn3c07669_si_001.pdf]

Supplementary Information

Development of Self-Assembled Protein Nanocage Spatially Functionalized with HA Stalk as a Broadly Cross-Reactive Influenza Vaccine Platform

Jaeyoung Park[a], *Julie A. Champion*[a,*]

[a]School of Chemical and Biomolecular Engineering, Georgia Institute of Technology, 950 Atlantic Dr. NW, Atlanta, GA, 30332-2000, USA.
[*]co-corresponding author: julie.champion@chbe.gatech.edu (J.A.C)

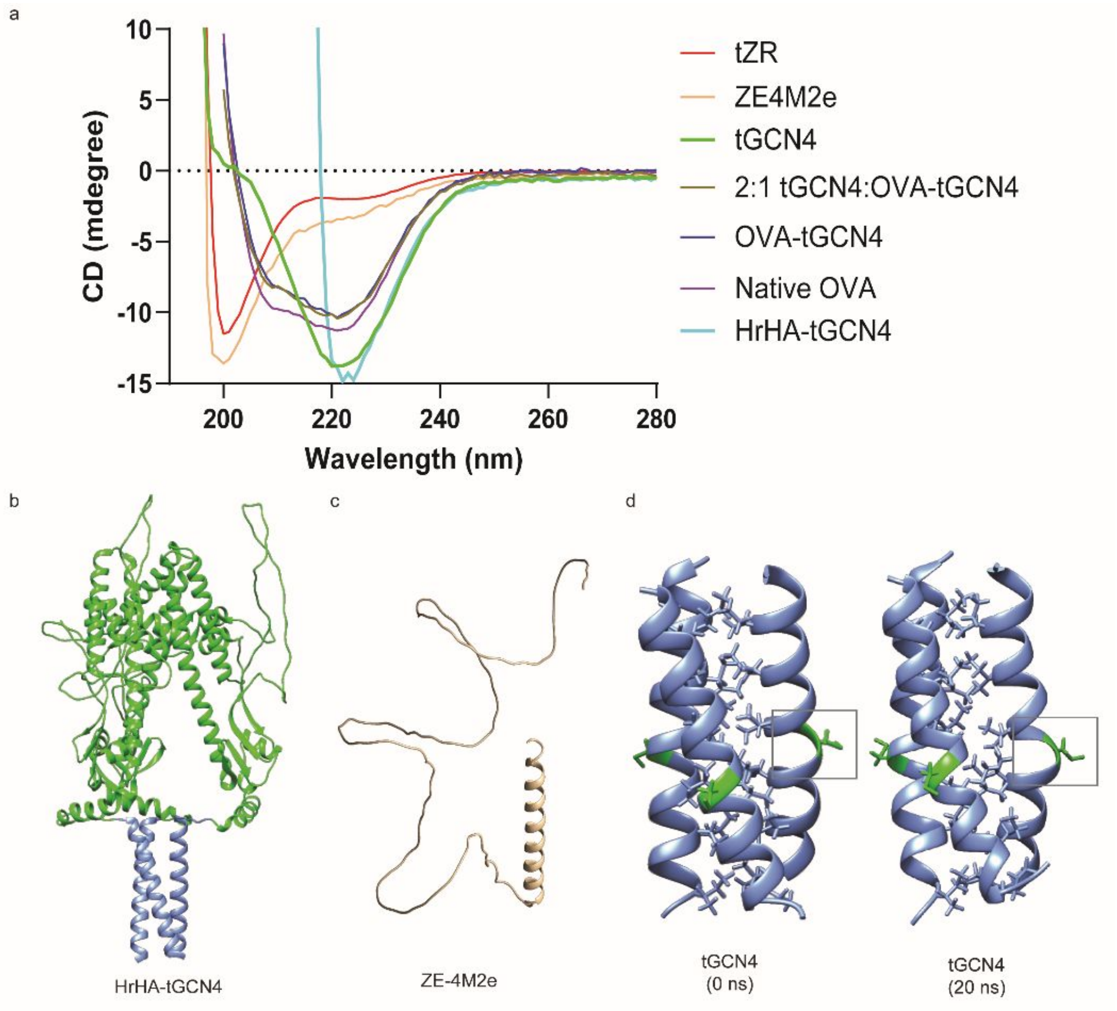

Figure S1. Assessment of SAPN modular protein structures. a Circular dichroism spectra of SAPN modular proteins (native OVA purchased commercially). *Ab initio* structures of **b** HrHA-tGCN4 and **c** ZE-4M2e computationally predicted by ColabFold. **d** Structural change in tGCN4 simulated by MD GROMACS for 20 ns.

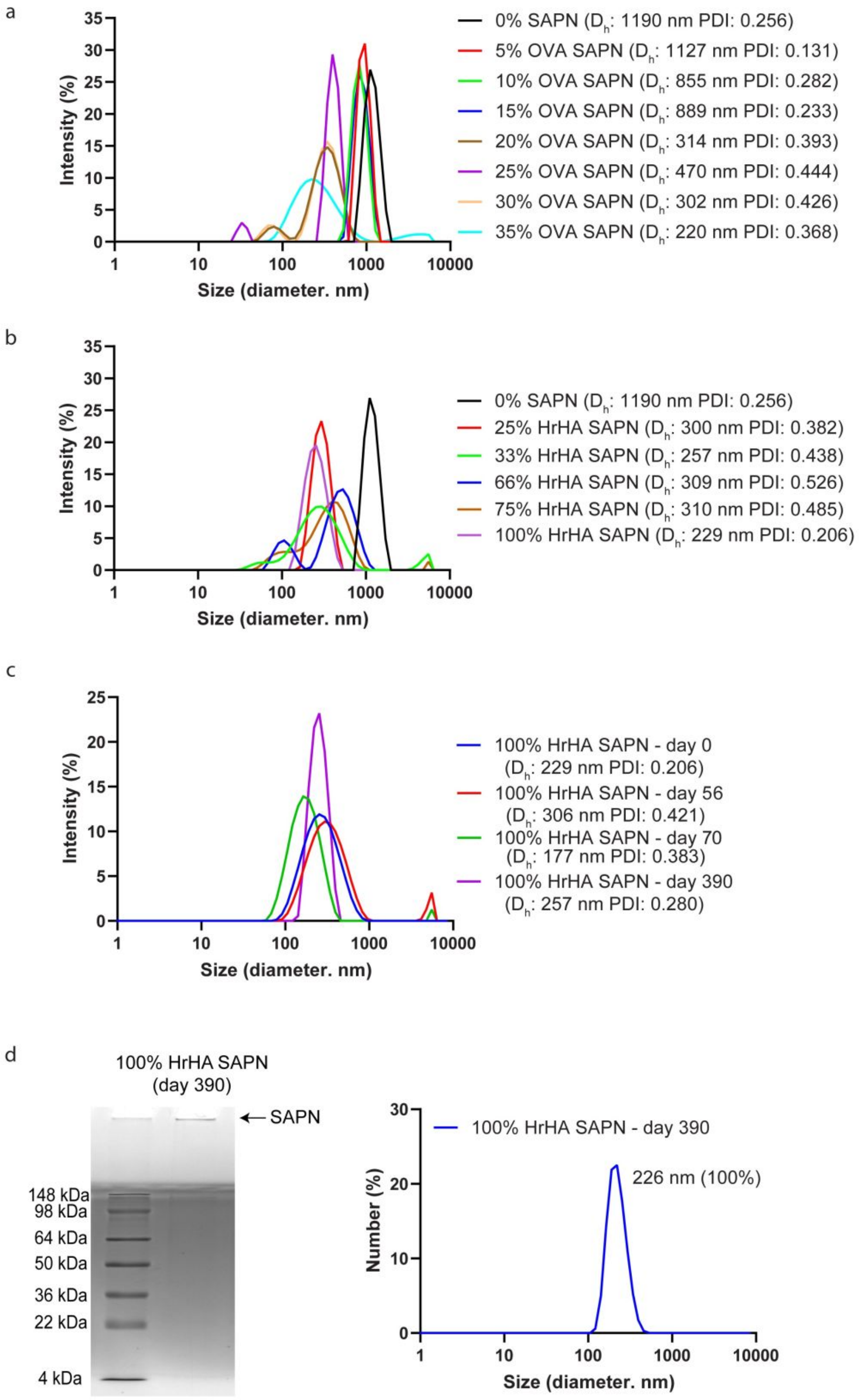

Figure S2. SAPN characterization. a OVA SAPN and **b** HrHA SAPN size distributions evaluated by DLS. **c** A time-course size measurement of 100% HrHA SAPN stored in PBS at 4°C by DLS. **d** SDS-PAGE gel electrophoresis separation (left) and a number-weighted size distribution (to better see small components from any disassembly) of 100% HrHA SAPN stored in PBS at 4°C for 390 days. Some aggregates were observed in 100% HrHA SAPN stored for 390 days; these were removed by centrifugation at 5,000 xg prior to analysis.

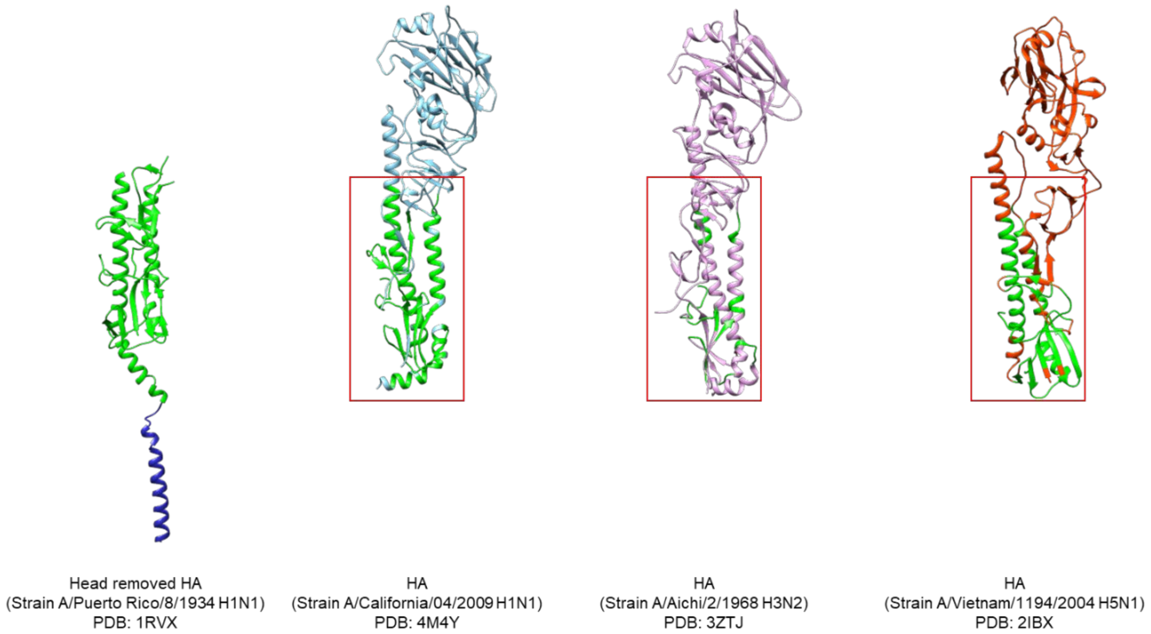

Figure S3. Sequence alignment of HA from different influenza A strains. The sequence of HrHA (green) derived from Strain A/Puerto Rico/8/1934 H1N1 fused to tGCN4 (dark blue) to make SAPN was compared to full HA sequences from A/California/04/2009 H1N1 (light blue; PDB: 4M4Y), A/Aichi/2/1968 H3N2 (pink; PDB: 3ZTJ), A/Vietnam/1194/2004 H5N1(red; PDB: 2IBX). Overlapped sequences are colored in green while each HA stalk is highlighted in a red box.

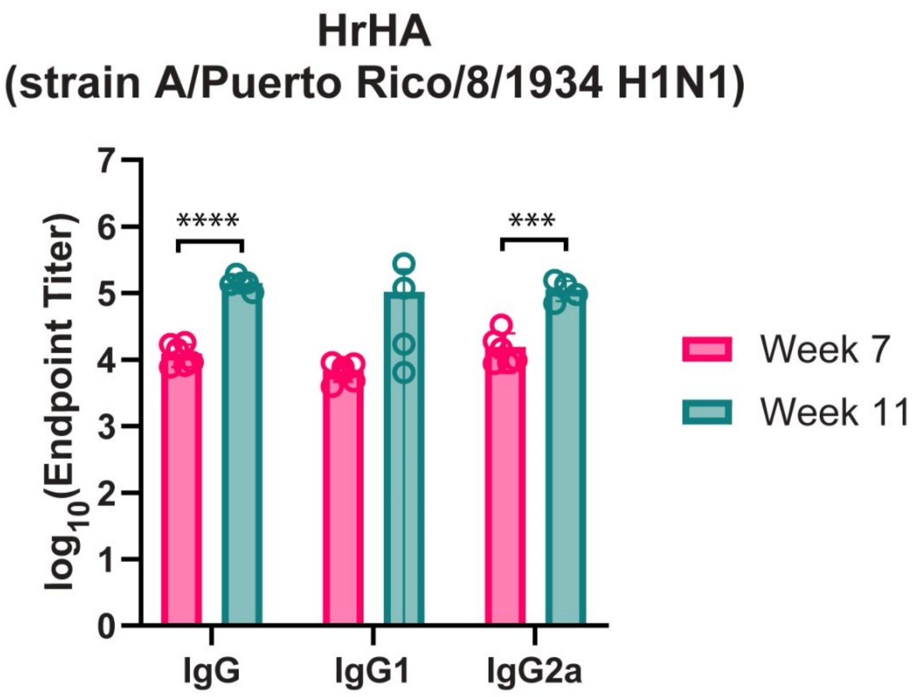

Figure S4. Anti-HrHA (Strain A/Puerto Rico/8/1934 H1N1) IgG endpoint titers collected (week 7 and week 11 post prime) from BALB/c mice vaccinated and boosted (week 4) with 100% HrHA SAPNs.

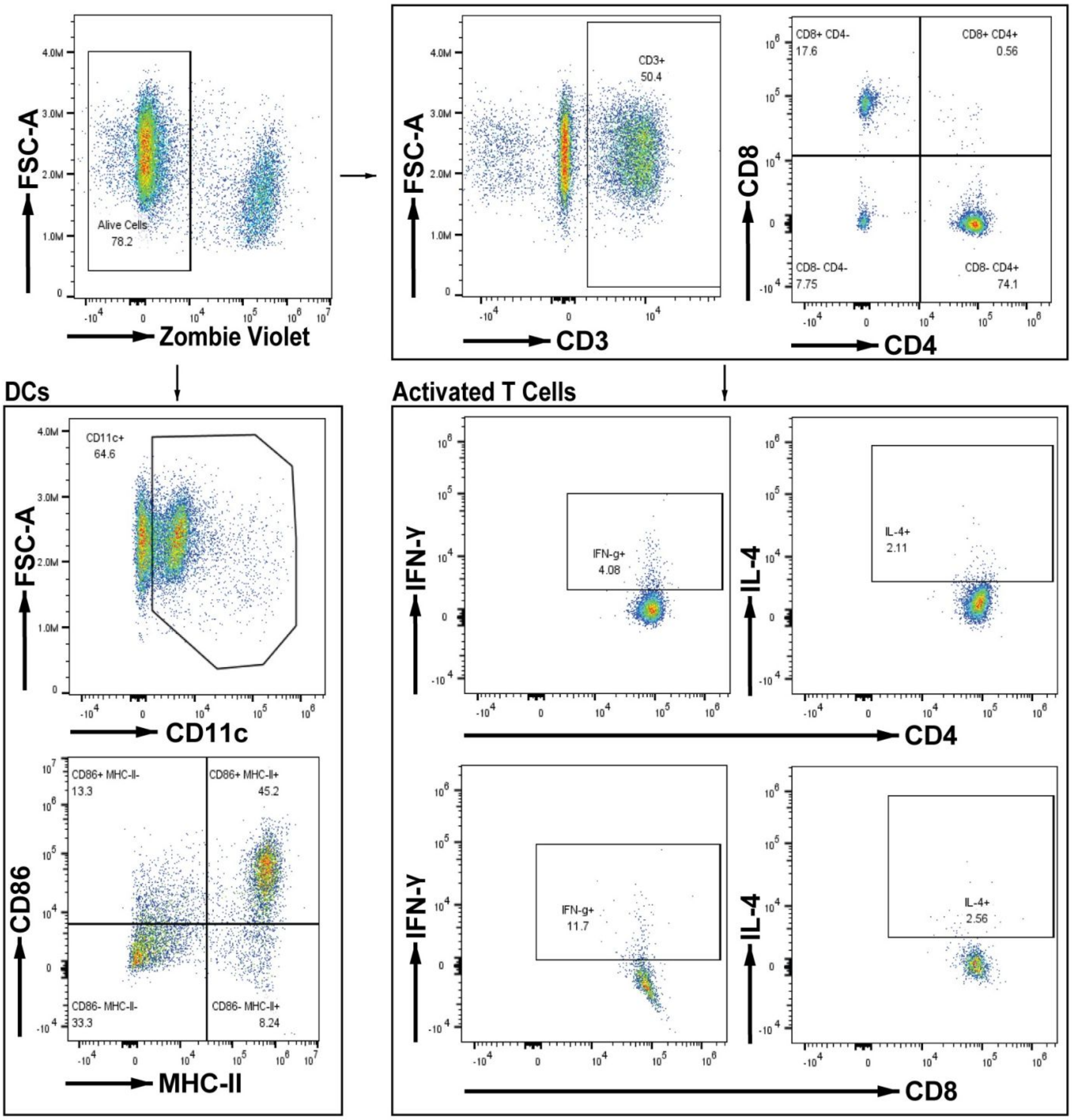

Figure S5. Gating strategy to identify activated DCs and T cell subsets.

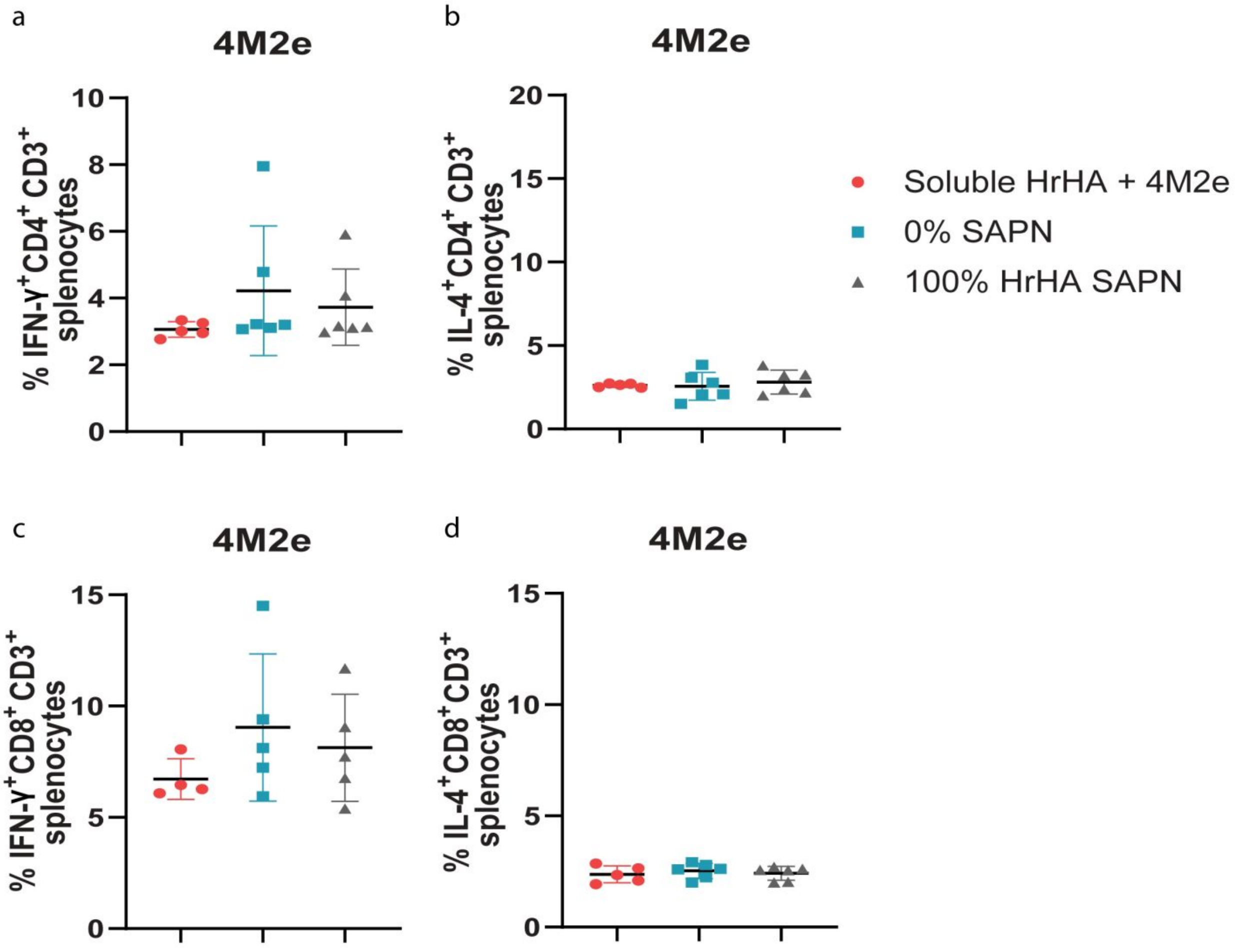

Figure S6. Cellular immune response of HrHA SAPNs immunized mice. Percentages of **a** IFN-γ^+ $CD4^+$ $CD3^+$, **b** IL-4^+ $CD4^+$ $CD3^+$, **c** IFN-γ^+ $CD8^+$ $CD3^+$, and **d** IL-4^+ $CD8^+$ $CD3^+$ T cells after restimulation by 4M2e antigens. The stained splenocytes were harvested from mice vaccinated with soluble HrHA+4M2e (red), 0% SAPNs (cyan), and 100% HrHA SAPNs (grey).

HrHA-tGCN4pII-His tag<br>(32 kDa)	MKANLLVLLCALAAADADTICIGYHANNSTDTVDTVLEKNVT<br>VTHSVNLLEDGGGGKYVRSAKLRMVTGLRNIPSIQSRGLFG<br>AIAGFIEGGWTGMIDGWYGYHHQNEQGSGYAADQKSTQNAI<br>NGITNKVNTVIEKMNIQGSGGSGDIWTYNAELLVLLENERTLD<br>FHDSNVKNLYEKVKSQLKNNAKEIGNGCFEFYHKCDNECM<br>ESVRNGTYDYPKYSEESKLNREKVDGVKLESMGIYVDPGSR<br>MKQIEDKIEEILCKIYHIENEIAGSGSENLYFQGSAGHHHHHH
His tag-OVA-tGCN4pII<br>(48 kDa)	HHHHHHSSGMGSIGAASMEFCFDVFKELKVHHANENIFYCP<br>IAIMSALAMVYLGAKDSTRTQINKVVRFDKLPGFGDSIEAQC<br>GTSVNVHSSLRDILNQITKPNDVYSFSLASRLYAEERYPILPE<br>YLQCVKELYRGGLEPINFQTAADQARELINSWVESQTNGIIRN<br>VLQPSSVDSQTAMVLVNAIVFKGLWEKAFKDEDTQAMPFR<br>VTEQESKPVQMMYQIGLFRVASMASEKMKILELPFASGTMS<br>MLVLLPDEVSGLEQLESIINFEKLTEWTSSNVMEERKIKVYL<br>PRMKMEEKYNLTSVLMAMGITDVFSSSANLSGISSAESLKIS<br>QAVHAAHAEINEAGREVVGSAEAGVDAASVSEEFRADHPF<br>LFCIKHIATNAVLFFGRCVSPGGSSGGSSENLYFQGRMKQIE<br>DKIEEILCKIYHIENEIA
ZE-4M2e-His tag (17 kDa)	EAAALEDENTALECEVAELEQEVQENLYFQGGSGGGSLLT<br>EVETPIRNEWGSRSNDSSDPGGSSGGSSSLLTEVETPTRS<br>EWESRSSDSSDPGGSSGGSSSLLTEVETPTRNGWESKSS<br>GSSDPGSGSGSGSSLLTEVETPTRNGWESNSSDSSDGSS<br>HHHHHH
tZR (3 kDa)	RAAALRDRNTALRCRVAELRQRVQ
tGCN4pII (3 kDa)	RMKQIEDKIEEILCKIYHIENEIA
HA (H1N1)-GCN4pII-<br>Avitag-His tag (66.94 kDa)	MKAILVVLLYTFATANADTLCIGYHANNSTDTVDTVLEKNVTV<br>THSVNLLEDKHNGKLCKLRGVAPLHLGKCNIAGWILGNPEC<br>ESLSTASSWSYIVETPSSDNGTCYPGDFIDYEELREQLSSVS<br>SFERFEIFPKTSSWPNHDSNKGVTAACPHAGAKSFYKNLIW<br>LVKKGNSYPKLSKSYINDKGKEVLVLWGIHHPSTSADQQSL<br>YQNADTYVFVGSSRYSKKFKPEIAIRPKVRDQEGRMNYYWT<br>LVEPGDKITFEATGNLVVPRYAFAMERNAGSGIIISDTPVHDC<br>NTTCQTPKGAINTSLPFQNIHPITIGKCPKYVKSTKLRLATGLR<br>NIPSIQSRGLFGAIAGFIEGGWTGMVDGWYGYHHQNEQGSG<br>YAADLKSTQNAIDEITNKVNSVIEKMNTQFTAVGKEFNHLEK<br>RIENLNKKVDDGFLDIWTYNAELLVLLENERTLDYHDSNVKN<br>LYEKVRSQLKNNAKEIGNGCFEFYHKCDNTCMESVKNGTYD<br>YPKYSEEAKLNREEIDGVPGSRMKQIEDKIEEILSKIYHIENEIA<br>RIKKLVGERGSGSENLYFQGSAGGLNDIFEAQKIEWHEGSG<br>SHHHHHH

Table S1. Protein sequences and molecular weight. HrHA-tGCN4 and H1N1 HA-GCN4 (used for ELISA assay) were expressed from Expi293F cells while tZE-4M2e and OVA-tGCN4 were expressed from *E. coli* BL21 Star (DE3). tZR and tGCN4 peptides were obtained by solid-phase synthesis (GenScript).
